# Discovery and Design of YTHDF2-Selective and Pan-YTHDF Inhibitors for m^6^A Binding by the YTH Aromatic Cage

**DOI:** 10.1021/acsomega.6c05268

**Published:** 2026-10-14

**Authors:** Vilbert Sikorski, Joni Salo, Simona Selberg, Diogo Costa Sa, Brunaldo Renzi, Indrek Teino, Daria Blokhina, Salla Virtanen, Lina Antenucci, Mart Saarma, Perttu Permi, Mati Karelson, Esko Kankuri

**Affiliations:** † Department of Pharmacology, 3835 University of Helsinki, Haartmaninkatu 8, 00290 Helsinki, Finland; ‡ Heart and Lung Center, Helsinki University Hospital, Haartmaninkatu 4, 00290 Helsinki, Finland; § Institute of Chemistry, 37546 University of Tartu, Ravila tn 14a, 50411 Tartu, Estonia; ∥ Chemestmed, Ltd, Soola tn 8, 51014 Tartu, Estonia; ⊥ Institute of Biotechnology, HiLIFE, University of Helsinki, Viikinkaari 5d, 00790 Helsinki, Finland; # Department of Chemistry, Nanoscience Center, University of Jyväskylä, Survontie 9 C, 40500 Jyväskylä, Finland; ∇ Department of Biological and Environmental Science, Nanoscience Center, University of Jyväskylä, Survontie 9 C, 40500 Jyväskylä, Finland

## Abstract

Recognition of N6-methyl-adenosine (m^6^A) in the RNA sequences by the YT521-B homology domain-containing protein family paralogs 1–3 (YTHDF1–3) is a dominant layer of myriad cellular regulation. Dysregulated YTHDF paralog expression and activities have been reported in cancer, including glioblastoma multiforme (GBM) in which YTHDF2 has been identified as a druggable dependency. Guided by structure-based *in silic*o screening, we identified structurally related small-molecule compound families of promising YTHDF inhibitor compounds. Following *in vitro* screening, two hits, DT11 and DT15, emerged and were further examined. We identified DT11 as a YTHDF2-selective inhibitor of the YTH domain using proximity-based bead assays, microscale thermophoresis, molecular dynamics, and nuclear magnetic resonance spectroscopy. DT11 demonstrated 10–100-fold YTHDF2-selectivity (IC_50_: YTHDF2 16.6 μM; YTHDF1 185 μM; YTHDF3 1300 μM) in inhibiting m^6^A-RNA binding, whereas the pan-inhibitor DT15 effectively inhibited all the paralogs (IC_50_: YTHDF2 12.6 μM; YTHDF1 18.8 μM; YTHDF3 31 μM). In LN229 glioblastoma cells, DT11 reduced cell viability and showed relevant additive efficacy with the gold standard Temozolomide. DT15 blocked GBM cell invasion and scratch wound closure. Similar effects were observed after inhibition of the m^6^A writer METTL3 activity by STM2457. No such effects were observed with DT11. A minutely modified DT11 derivative, DT11-1, showed 10-fold increases in YTHDF2-selective m^6^A binding and LN229 cell viability inhibitory potencies. All the compounds produced high GBM preference over primary neurons and HEK293 cells in their effects. Our results further define pharmacological molecular comprehensions for paralog-dependent engagement of m^6^A in RNA by the aromatic cage for the selective modulation of YTHDF functions.

## Graphical abstract

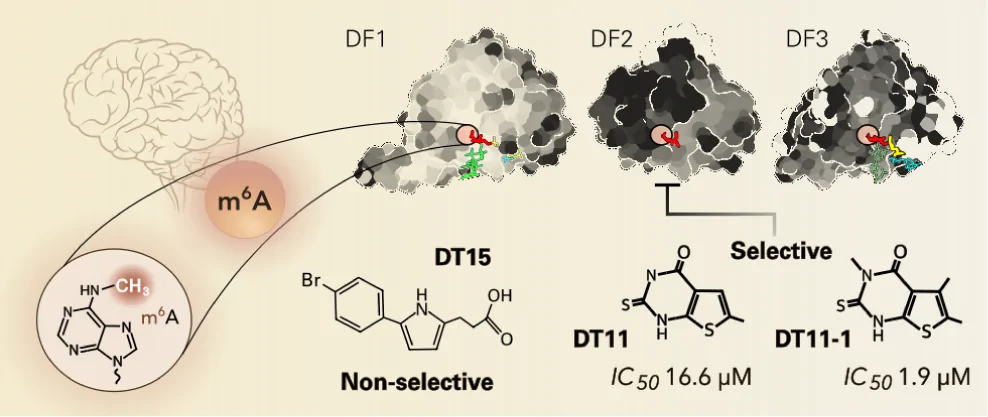

## Introduction

RNA transduces the genomic information in all cells. Among the over 300 currently identified post-transcriptional modifications of RNA bases,^1^ the reversible *N* ^6^-methyladenosine (m^6^A) regulates the stability and translation of tens of thousands of RNAs.^2–4^ The YT521-B homology domain-containing protein family paralogs 1–3 (YTHDF1–3) that bind m^6^A via an aromatic cage in the conserved C-terminal YTH-domain have emerged as key readers of m^6^A on RNAs.^5,6^ The extent of YTHDF paralogs’ redundant and distinct functions remains debated, with recent reviews and experiments suggesting shared cage recognition but with divergent N-terminal disordered domain behaviors for the context-dependent downstream functions.^6–10^ Targeting the YTHDF paralogs’ aromatic m^6^A binding cage for their functional antagonism is supported by crystal structures showing the m^6^A in RNA to be captured by a tryptophan-lined pocket and neighboring acidic and polar residues.^11–13^ This aromatic cage has recently emerged as a promising target for competitive small-molecule antagonism to modulate m^6^A-dependent RNA-YTHDF interactions.^6,14–18^

Glioblastoma multiforme (GBM), the most common, aggressive, and deadly of primary malignant formations of the adult brain,^19^ is characterized by post-transcriptional dysregulation^20^ and, more recently, by specific dysregulation of m^6^A-containing long noncoding RNAs (lncRNAs)^21^ and mRNA subsets.^22^ A recent preprint described the first direct RNA nanopore sequencing of patient-derived GBMs for m^6^A maps across isocitrate dehydrogenase 1 (IDH1) mutant and wild-type GBMs to find the mutants with high m^6^A content, increased expression of m^6^A writers, and m^6^A-RNA stabilizing YTHDF3, while the wild types had elevated m^6^A erasers and the primarily m^6^A-RNA-decay-linked YTHDF2.^23^ GBM invades aggressively, and often acquires resistance to drugs, such as Temozolomide (TMZ).^24^ The role of YTHDF2 in this aggressiveness^25^ and TMZ resistance^32^ supports the rationale for developing small-molecule inhibitors that target it selectively. Indeed, recent data by Radhi et al. found that the m^6^A-RNAs in GBM tumors were significantly more abundant in the rim and invasive parts of the tumor.^26^

Here we report the discovery of DT11 (YTHDF2-selective) and DT15 (pan-YTHDF) compounds, which inhibit the YTH-domains’ binding to m^6^A-modified RNA (m^6^A-RNA). These compounds show preferential efficacy in LN229 GBM cells over HEK293 cells and primary cortical neurons, and DT11 demonstrates relevant additive effects when coadministered with TMZ. Together, our results help to define YTHDF paralog-dependent pharmacology for the m^6^A cage binding inhibition by providing molecular and cellular insights fit to pave the way toward next-generation ligands with still improved selectivity and potency.

## Results

### The YTHDF YTH-Domains Bind m^6^A RNA with High Selectivity over Native, Nonmethylated RNA

The binding selectivity of the human YTHDF1–3 YTH-domains for m^6^A was evaluated by cross-comparing biotinylated m^6^A-modified 27-nucleotide RNA (B-m^6^A-RNA) over its native, nonmethylated identical counterpart (B-A-RNA), both of which contained the DRACH consensus motif (D = A/G/U, R = A/G, H = A/C/U) for the major m^6^A methyltransferase complex (MTC).^27^ Based on the screening matrices for optimal signal-to-noise binding concentrations, the protein concentrations (YTHDF1, 100 nM; YTHDF2, 125 nM; YTHDF3, 275 nM) for the assay were based on the observed maximal signal with B-m^6^A-RNA over that of B-A-RNA (48 nM for YTHDF1 and 24 nM for YTHDF2–3) with care to not exceed the assay hook points (Figure 1A–C, left panels). The selectivity of the paralogs for m^6^A binding over nonmethylated oligo was 9.1-fold and 6.3-fold, in the AlphaScreen assay for YTHDF1 and YTHDF2, respectively. The nonexistent B-A-RNA binding compared to bead control for YTHDF3 prevented calculation of a meaningful selectivity fold change.

**Figure 1:**
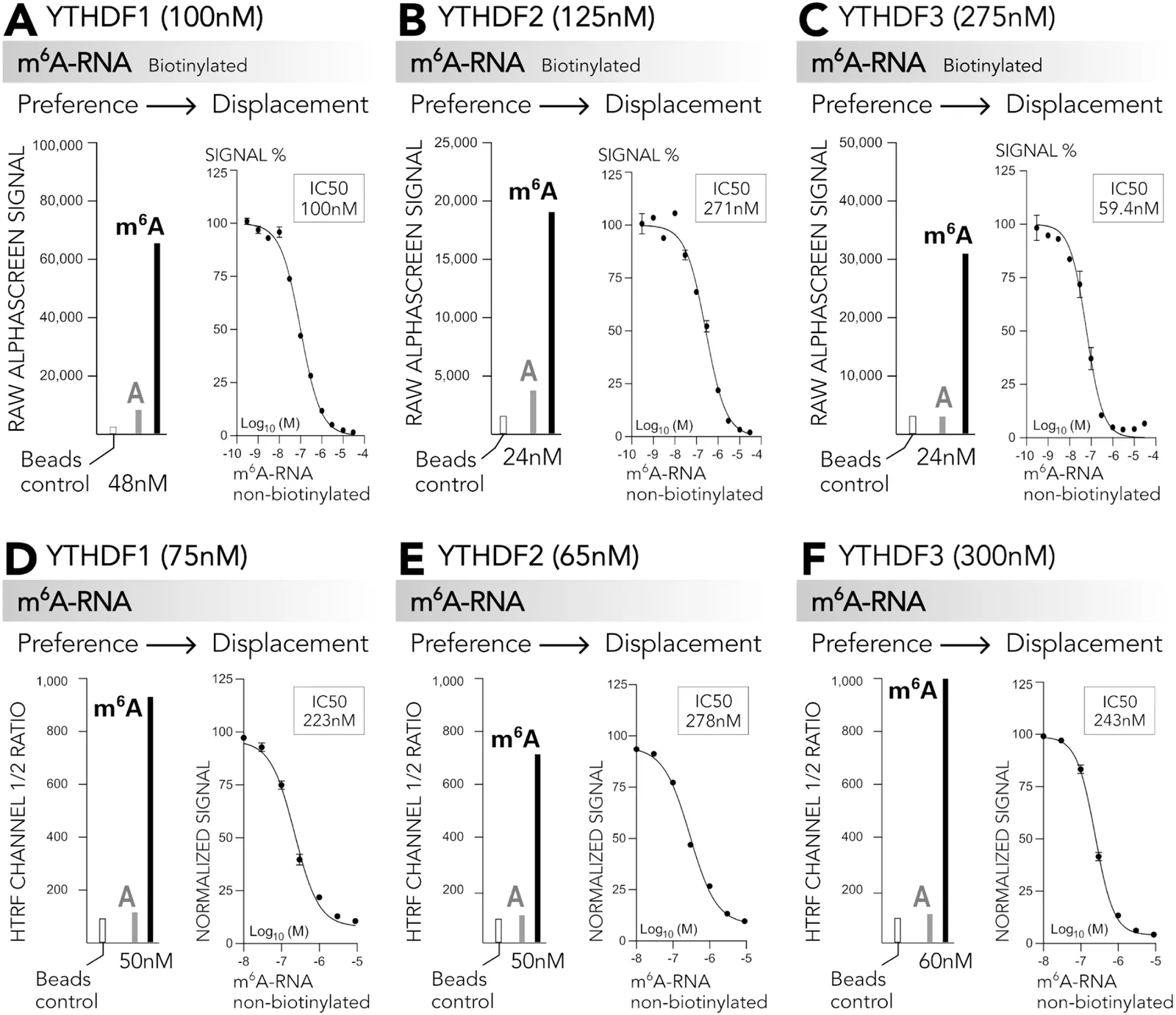
Binding preferences for m^6^A-RNA over A-RNA and displacement of YTHDF1–3 YTH-domains’ binding to biotinylated m^6^A-RNA by identical, nonbiotinylated m^6^A-RNA. In the AlphaScreen assay (A–C), the m^6^A binding preferences of YTHDF YTH-domain protein constructs are observed. On the right in each panel, the displacement assay unraveled the assay’s dynamic range, validated the nanomolar-range potency of m^6^A to bind its pocket, and laid the base for subsequent inhibitor screening. A homogeneous time-resolved fluorescence (HTRF) setup (D–F) was used to validate the hit inhibitors for false positives. In displacement assays, all data points represented technical triplicates from one experiment. All paralogs were examined in tandem to exclude batch effects. For m^6^A binding preferences, the signals are representative results for each paralog and the used method.

To validate the assay for inhibitor screening, we assessed the competitive efficacy of a nonbiotinylated identical m^6^A-RNA (i.e., Comp-m^6^A-RNA) to inhibit B-m^6^A-RNA binding and assay signal generation. Expectedly, Comp-m^6^A-RNA inhibited B-m^6^A-RNA-dependent signal at nanomolar half-maximal inhibitory concentrations (IC_50_), for YTHDF1 100 nM, YTHDF2 271 nM, and YTHDF3 59.4 nM (Figure 1A–C, right panels), thus validating the method to screen for inhibitors at m^6^A binding aromatic pocket.

To enable validation using an orthogonal method, we established a second *in vitro* assay based on homogeneous time-resolved fluorescence (HTRF) bead chemistry. The assay reproduced the expected m^6^A-binding preference of the YTHDF proteins (Figure 1D–F, left) and demonstrated nanomolar-level competitive inhibition by the nonbiotinylated m^6^A ligand with IC_50_ values of 223 nM for YTHDF1, 278 nM for YTHDF2, and 243 nM for YTHDF3 (Figure 1D–F, right).

### Molecular Docking for Human YTHDF1–3 YTH-Domains Reveals a Set of Distinct Compounds Predicted to Inhibit m^6^A Binding in RNA

To examine the ligand–protein interactions involved within YTHDF1–3 m^6^A binding inhibition, molecular docking was carried out using Glide^28–30^ on nearly 140,000 small molecules from the FIMM database. Calculated binding-pose analysis revealed 79 compounds with high docking-efficiency predictions for binding to the aromatic m^6^A pocket site of YTHDF1–3 similar to m^6^A as reported based on the X-ray crystal structures of the proteins in complex with m^6^A-RNA.^11–13^ The binding pockets of several reported inhibitors of YTHDF family proteins at this pocket bear much similarities across the YTHDF paralogs.^16–18^ Based on both the docking results and commercial availability, 10 active compounds belonging to different scaffolds were identified as suitable ligands for these proteins.

For all three proteins and 10 experimentally active compounds in docking, the most important interaction was a π–π interaction between the phenyl or heterocyclic group of the ligand and a tryptophan residue of the protein, i.e., Trp470 in YTHDF1, Trp491 in YTHDF2, and Trp497 in YTHDF3. Furthermore, hydrogen bonds between the ligands and proteins also contributed significantly to the docking efficiency. In the case of YTHDF1, the residues Lys395, Tyr397, Asp401, Cys412, Arg506, and Asp507 were found to be important in the docking of the ligands. For the YTHDF2 protein, hydrogen bonding between the ligand and the residues Lys416, Tyr418, Asp422, Cys433, Asn462, and Asp528 played a significant role. In the docking of ligands to the YTHDF3 protein, hydrogen bonds with the residues Asp534, Tyr424, Asp428, and Cys439 were often noted.

### Screening of DT4 and DT13 and Their Derivatives Reveals a Loss of Initial Potency and Limited Paralog Selectivity in Inhibiting m^6^A Binding by YTHDF1–3

Based on the molecular docking results, we first evaluated the effects of compounds DT13 and DT4. Both compounds (Figure 2A,G) inhibited B-m^6^A-RNA binding to the YTH-domains with low micromolar potencies: DT13 with IC_50_ values of 17.0 μM, 10.5 μM, and 49.4 μM for YTHDF1, YTHDF2, and YTHDF3, respectively, and DT4 with IC_50_ values of 7.84 μM, 10.1 μM, and 16.7 μM for YTHDF1, YTHDF2, and YTHDF3, respectively. Based on these results, we designed and synthesized a set of compound derivatives, five for DT13 and two for DT4. Only DT13–3 showed little increase in selectivity for YTHDF2 (Figure 2D). The other derivatives for DT13 and DT4 demonstrated no activity to inhibit YTH-domain binding to B-m^6^A-RNA in the AlphaScreen assay.

**Figure 2:**
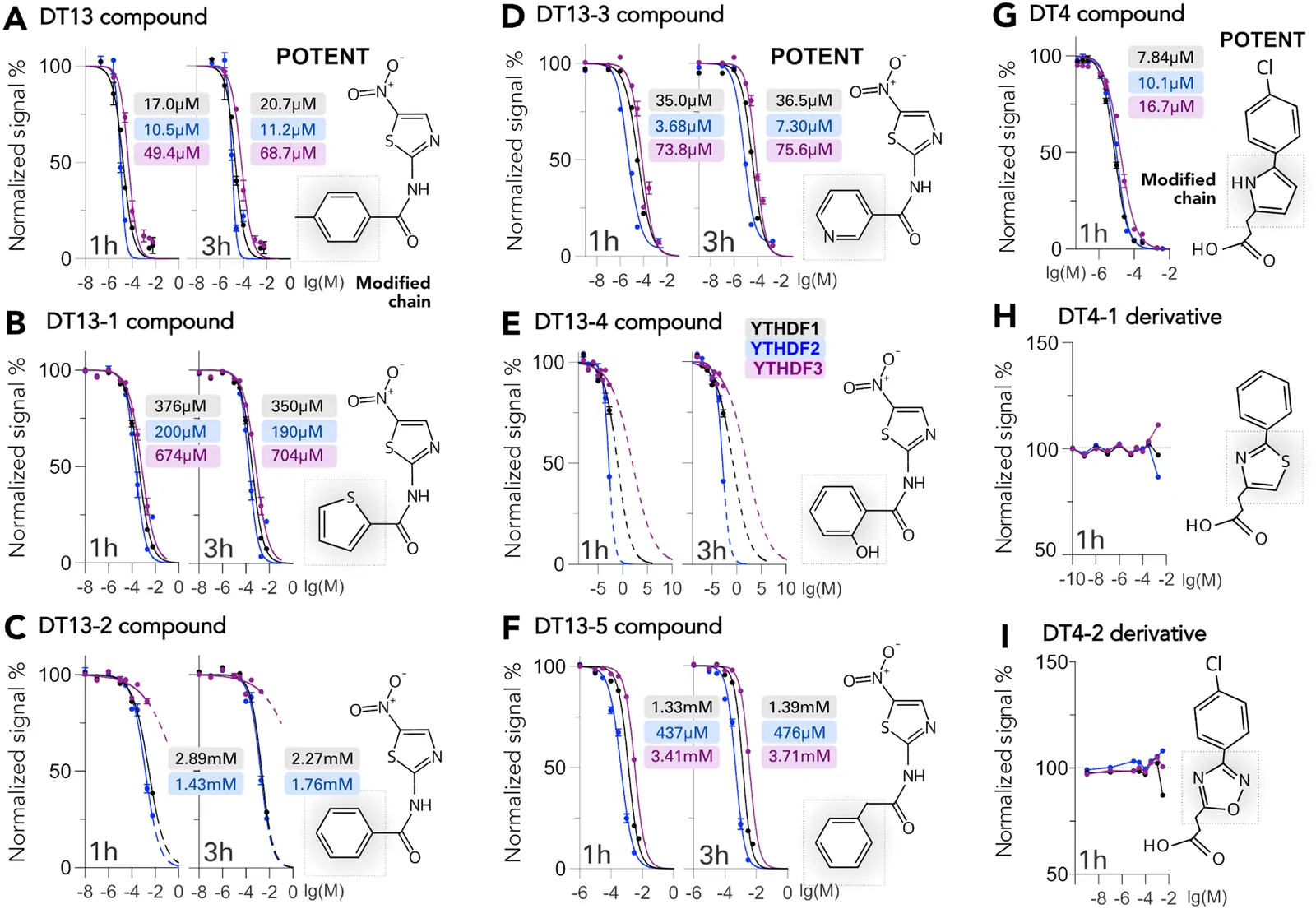
Identification of DT13 and DT4 compounds as pan-paralog inhibitors of m^6^A binding in RNA for YTHDF1–3 with lost efficacies for most of their derivatives. (A) DT13 compound structure and potency to inhibit binding m^6^A on RNA for YTHDF1–3 YTH-domains. IC_50_ values are shown with colors corresponding to each paralog. The initial selectivity of the DT13 compound was selected to be explored with derivatives: (B) DT13–1, (C) DT13–2, (D) DT13–3, (E) DT13–4, and (F) DT13–5. Of these compounds, only DT13–3 demonstrated signs of enhanced potency with moderate 5–10-fold selectivity. (G) For the DT4 compound with relatively low micromolar IC_50_ values, the derivatives DT4–1 and DT4–2 both demonstrated full loss of efficacy (H, I). Each data point represents five to six technical replicates pooled from two experiments (plates). Data are shown as mean ± SEM. IC_50_, half maximal inhibitory concentration. IC_50_ values are shown with colors corresponding to each paralog.

### Identification of the YTHDF2-Selective Inhibitor DT11 and the Pan-YTHDF Inhibitor DT15

In further evaluation of compounds in the AlphaScreen assay based on the molecular docking results (Figure 3), we found two compounds with low-micromolar efficacy, DT11 and DT15 (Figure 3C,D). DT11 inhibited B-m^6^A-RNA binding to the YTH-domain of YTHDF2 with up to 100-fold selectivity over that of YTHDF3 and 10-fold selectivity over the YTH-domain of YTHDF1 (IC_50_ values 185 μM, 16.6 μM, and 1.3 mM for YTHDF1, YTHDF2, and YTHDF3, respectively). DT15 inhibited B-m^6^A-RNA binding across all paralogs’ YTH-domains (IC_50_ 18.8 μM, 12.6 μM, and 31 μM for YTHDF1, YTHDF2, and YTHDF3, respectively) without such differences. DT11 was designated as a YTHDF2-selective inhibitor and DT15 as a pan-YTHDF inhibitor. DT11 and DT15 were next tested with the biotinylated-FLAG control reagent and the binding beads at concentrations corresponding to their IC_50_s. The compounds did not significantly affect the signal in this control system. At 15 μM of DT15, this signal was reduced just by 10%, while DT11 at 15 μM actually minutely increased the signal (∼5%).

**Figure 3:**
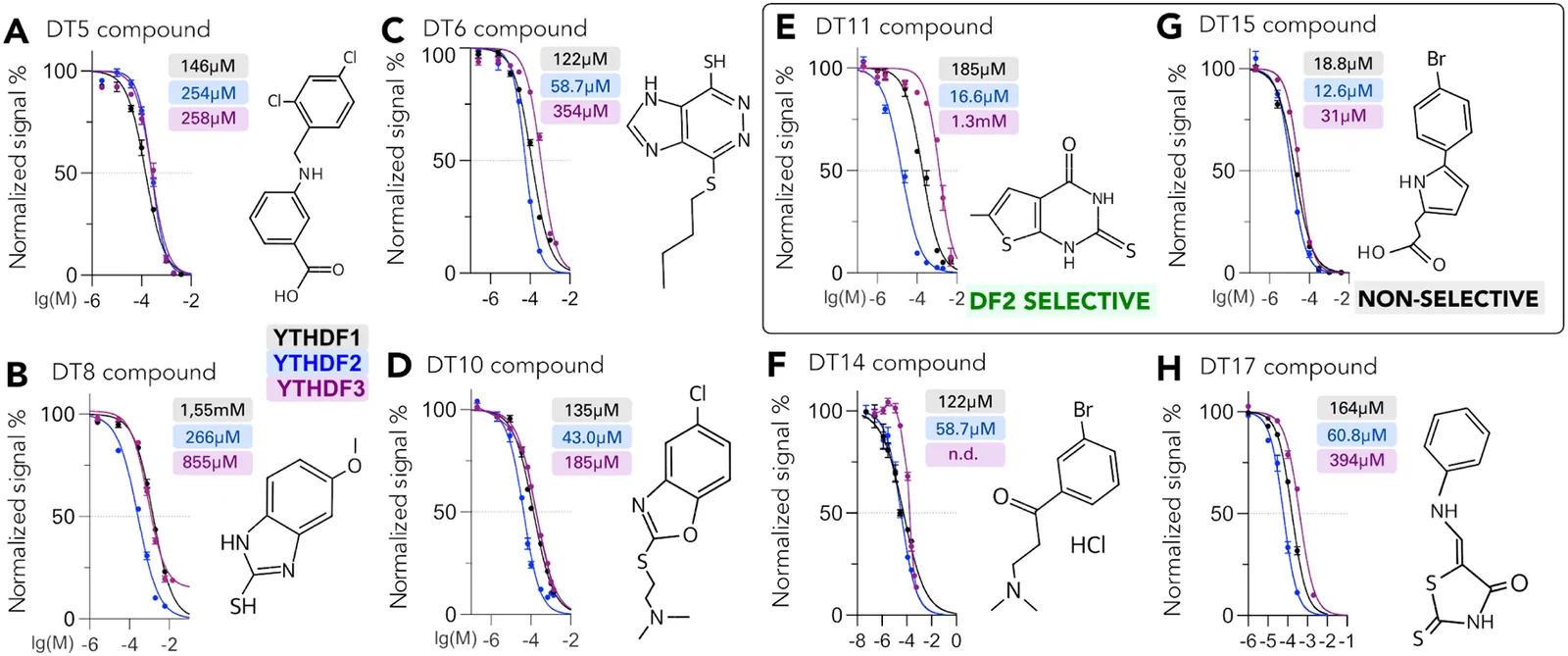
Further screening reveals compound DT11 with up to 100-fold selectivity toward m^6^A in RNA binding inhibition of YTHDF2 and DT15 with comparable potency as an unselective inhibitor. Based on further compound screening, we identified inhibitors (A) DT8 and (B) DT10 with a trend for YTHDF2 selectivity, while testing of (C) DT11 compound revealed superior, 10- and 100-fold selectivity of m^6^A binding inhibition for YTHDF2 over that of YTHDF1 and YTHDF3, respectively. The screening also identified the nonselective (E) DT5 compound with moderate micromolar IC_*5*0_ values, and (D) DT15 compound with low micromolar inhibition across all YTHDF paralogs. The chemical compound scaffolds (F) DT6, (G) DT14, and (H) DT17 did not reveal greater potencies than delivered by DT15. Each data point represents five to six technical replicates pooled from two experiments (plates). Data are shown as mean ± SEM. IC_50_ values are shown with colors corresponding to each paralog. IC_50_, half maximal inhibitory concentration.

### Molecular Dynamics of DT11 and DT15 Binding to YTHDF1–3 YTH-Domains Supports the YTHDF2 Selectivity of DT11 and Nonselectivity of DT15

According to the *in silico* molecular dynamics simulation results, compound DT11 forms a hydrogen bond with the Cys433 residue of the YTHDF2 protein, which remains stable throughout the simulations (Figure 4, middle panels). Additionally, a stronger water bridge is suggested with an aspartic acid located in the active center. In contrast, no such long-persisting strong hydrophobic interactions were found for the other paralogs, while for YTHDF3 the simulations showed that the arrangement of its active site differed, reducing the robustness of direct comparisons. However, for YTHDF3, compared to YTHDF2, there were significantly fewer interactions. It is suggested by the simulations that hydrophobic interactions play a crucial role in the binding of compound DT11 to the proteins. For DT15, the simulations exhibited comparable interaction profiles without significant differences across all three YTHDF YTH-domains. These results corroborate experimental data to support the conclusion that compound DT15 is a nonselective inhibitor of m^6^A binding by the human YTH-domain’s aromatic cage, while DT11 is a selective one.

**Figure 4:**
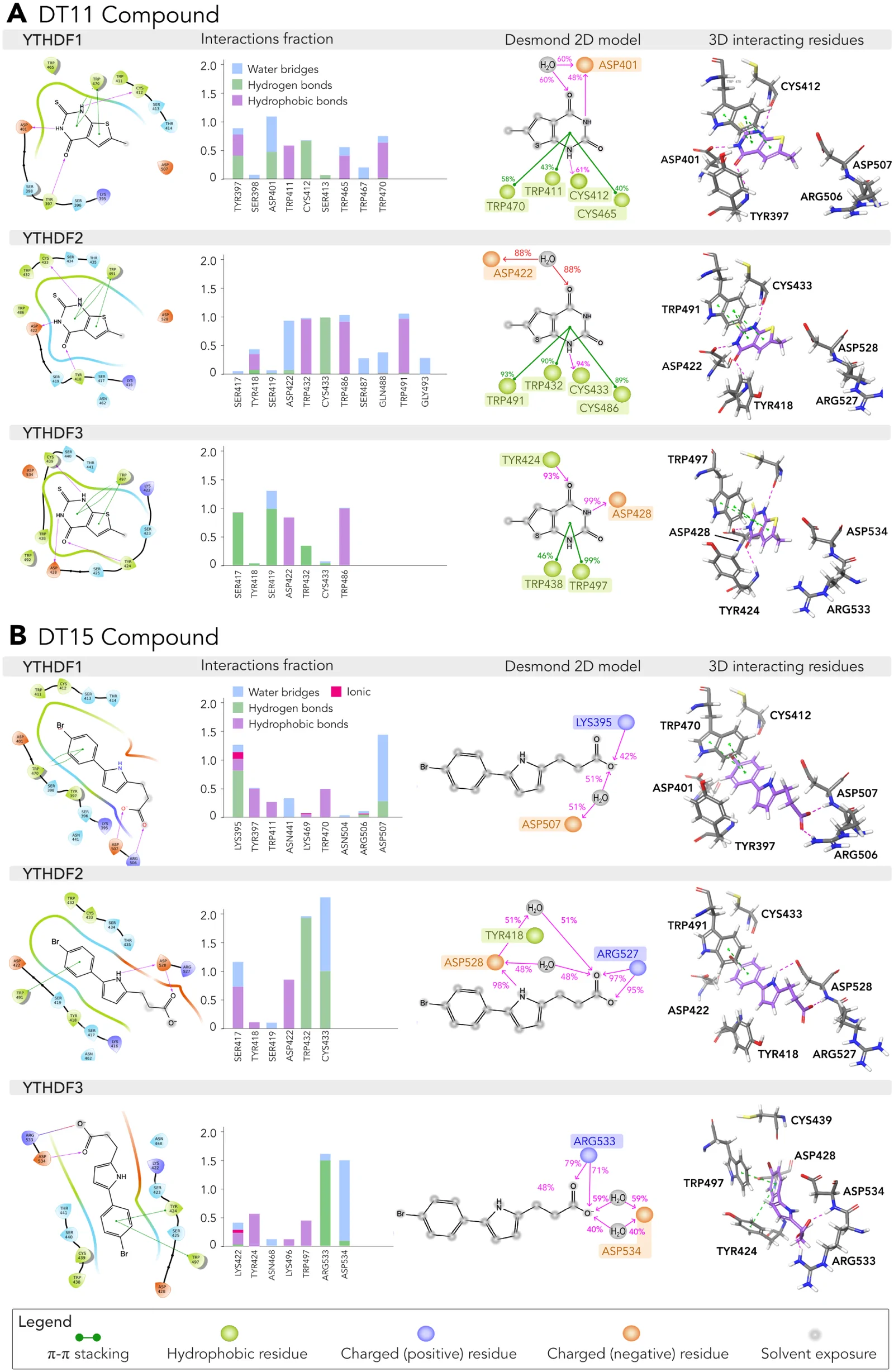
Molecular docking and dynamics simulations of YTHDF paralogs with (A) DT11 and (B) DT15 during 10 ns simulations. On the left side are shown the docking binding positions of DT11 and DT15 at the orthosteric aromatic cage in available YTHDF1 (PDB: 4RCJ),^11^ YTHDF2 (PDB: 4RDN),^12^ and YTHDF3 (PDB: 6ZOT)^13^ crystals. In the middle, the normalized stacked bar chart of interactions and contacts between the protein and ligand over the course of the simulation trajectory; interactions occurring above 40% of the simulation are shown. On the right side of the panels are shown Desmond 2D profiles and 3D models of the interacting residues based on the simulations.

### Microscale Thermophoresis (MST) and HTRF Validate the DT11 and DT15 Interactions with YTHDF1–3 YTH-Domains with and without m^6^A

In the MST assay, DT11 *K*_d_ for YTHDF2 was found as 18.4 μM (Figure 5), which corroborated its IC_50_ of 16.6 μM for m^6^A binding inhibition measured by the AlphaScreen assay. Interestingly, for YTHDF3, the *K*_d_ was just 8.0 μM, while the IC_50_ was as high as 1.3 mM, which suggests binding can occur also elsewhere other than in the m^6^A binding aromatic cage. However, the lack of a *K*_d_ for YTHDF1 suggested either no binding or binding uncaptured by the method. However, specifically at the highest tested concentrations, a tendency for a binding signal could be seen. We then verified the m^6^A-RNA interaction with YTHDF2 to be considerably more potent (*K*_d_ of 1.8 μM) than that of DT11 (Figure 5B), aligning with the IC_*5*0_ values for the nonbiotinylated m^6^A-RNA displacement and DT11 AlphaScreen results. One hour preincubation of YTHDF paralogs with DT11 abolished the binding signals for m^6^A-RNA (Figure 5C), suggesting that DT11 binding affects the aromatic m^6^A binding cage’s capacity to bind m^6^A on RNA. We also established the interactions of DT15, which, again in some discordance with the low micromolar IC_50_ values, displayed moderate micromolar to millimolar *K*_d_ values (YTHDF1: 453 μM, YTHDF2: 217 μM, YTHDF3: 2.1 mM). Finally, we validated the m^6^A binding inhibition of DT11 selectivity and DT15 nonselectivity by HTRF with the identical proteins and m^6^A-RNA (Figure 5E,F).

**Figure 5:**
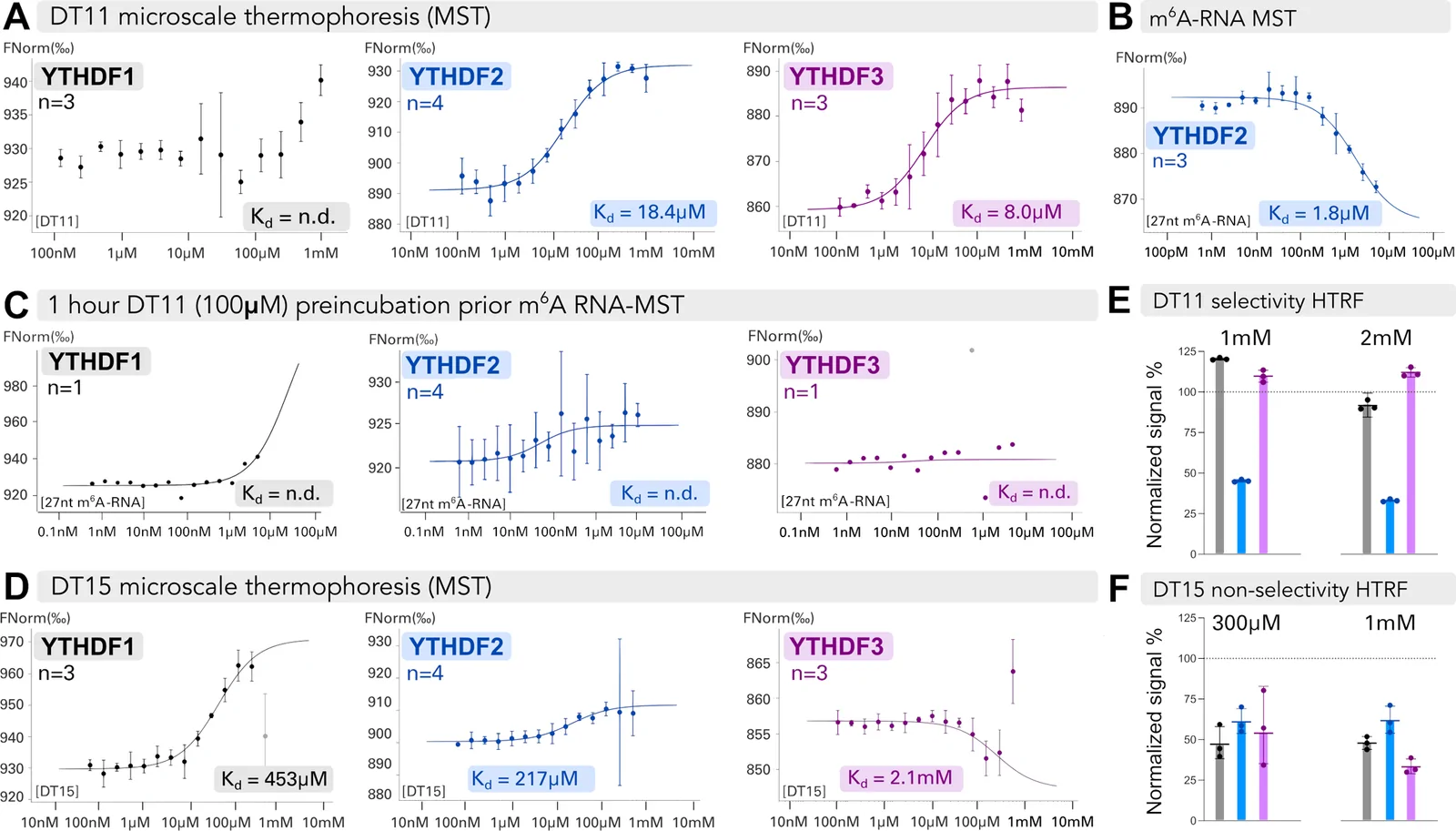
Microscale thermophoresis (MST) and homogeneous time-resolved fluorescence (HTRF) of YTHDF1–3 with DT11 and DT15 compounds. **(**A**)** DT11 binding is established by MST for YTHDF2 and YTHDF3, while no clear curvature is detected for YTHDF1. The *K*_d_ value of 18.4 μM for YTHDF2 corroborates well with the IC_50_ for m^6^A binding inhibition (16.6 μM, Figure 3), but *K*_d_ 8.0 μM for YTHDF3 and IC_50_ 1.3 mM differ distinctly. (B) MST revealed a high m^6^A binding potency (*K*_d_ 1.8 μM). (C) In agreement with m^6^A binding inhibition (Figure 3), preincubation of YTH-domains with DT11 blocked the m^6^A-RNA binding curve, supporting that DT11 inhibits m^6^A binding by binding to YTH-domains. (D) DT15 binds all the YTHDF paralogs. The DT11 selectivity (E) and DT15 nonselectivity (F) in inhibiting m^6^A binding by YTHDF1–3 YTH-domains were validated in the HTRF assay. Data are presented as mean ± SEM, and the number of replicates is shown in the subplots. For (A–D), these represent biological replicates, while for (E, F) these represent technical replicates.

### Nuclear Magnetic Resonance (NMR) Spectroscopy Validates DT11 Binding to the YTHDF2 YTH-Domain

Solution-state NMR spectroscopy was employed to observe the direct binding between DT11 and YTHDF2. To this end, we expressed and purified the YTH-domain of YTHDF2, residues 401–554, as an ^15^N-labeled protein. Figure 6 showcases the two-dimensional ^15^N,^1^H HSQC spectrum of free YTHDF2 (red contours) overlaid with YTHDF2 spectra after titrating 16× and 32× excess of nonlabeled DT11 (blue and green contours). Binding-induced chemical shift perturbations (CSPs) upon the addition of DT11 were observed for several residues in a dose-dependent manner, supporting the presence of a direct interaction between YTHDF2 and DT11. However, a 64-fold excess of the compound induced more pronounced CSPs, affecting approximately half of the recorded peaks and indicating possible phase separation or oligomerization effects rather than a specific interaction (Figure S1).

**Figure 6:**
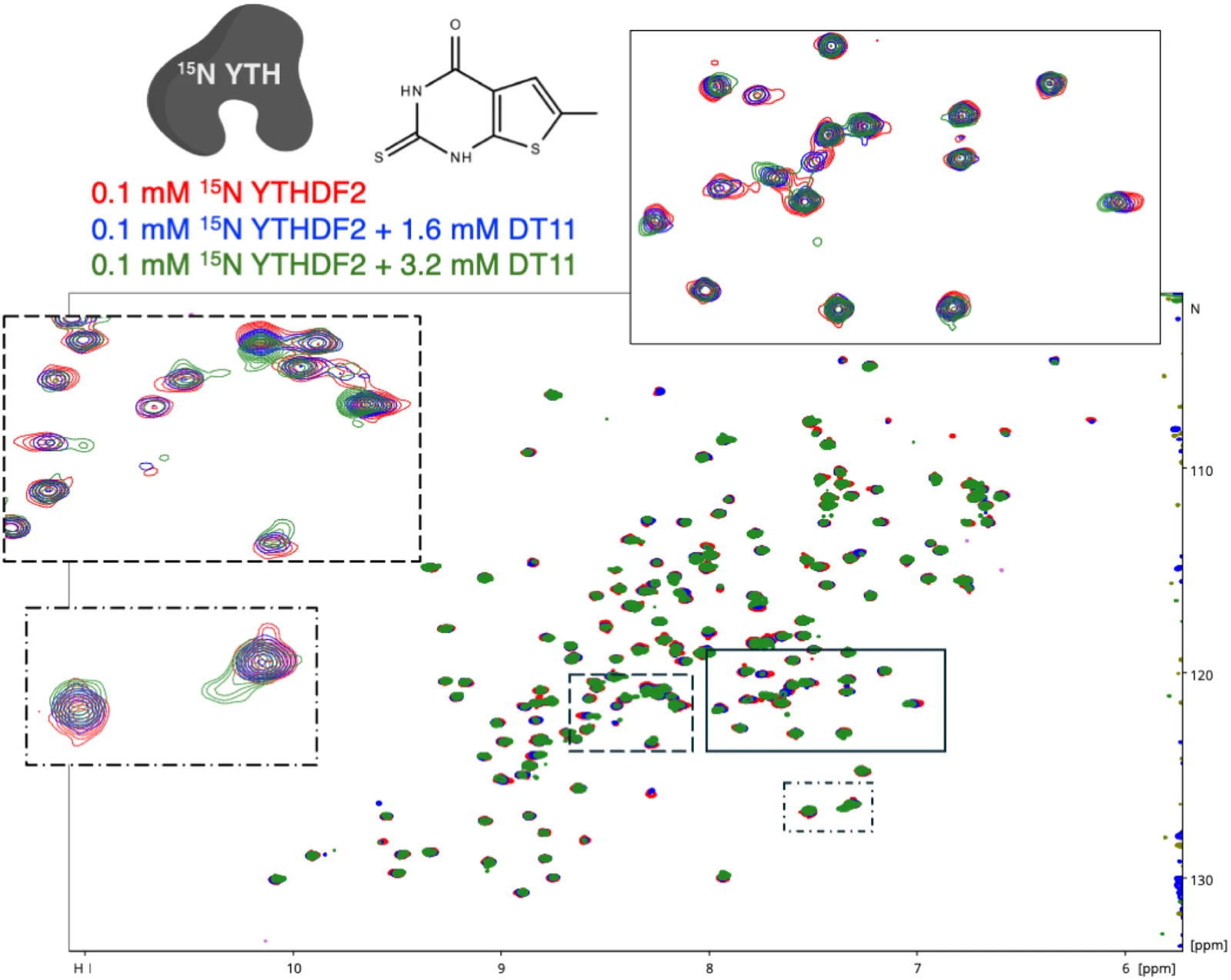
Binding-induced chemical shift perturbations on ^15^N YTHDF2 m^6^A binding YTH-domain upon addition of DT11. ^15^N-HSQC spectra of free 0.1 mM ^15^N-labeled YTHDF2 (red), and in the presence of 1.6 mM (blue) and 3.2 mM (green) of unlabeled DT11, are shown overlaid. Three insets, indicated by solid and dashed line rectangles, show expansions from selected spectral regions, where significant CSPs can be observed in a dose-dependent manner in several of the peaks, agreeing with the signal seen as the compound binds the target. The protein icon was created using BioRender.com.

### DT11 and DT15 Inhibit Glioblastoma LN229 Cell Line Viability, but Only DT15 Prevents Their Invasion and Scratch Wound Closure

In the PrestoBlue cell viability assays of LN229 GBM cell responses, both DT11 and DT15 showed comparable inhibitory potencies, yet also notable differences. Experiments were carried out in low (3%) and high (10%) fetal bovine serum (FBS) concentrations to evaluate the cells’ effects to the compounds under varying mitotic stimuli. As shown in Figure 7A, DT11 inhibited LN229 cell viability at 2 days without significant differences across mitotic stimulus levels (3% FBS, IC_50_ 318 μM; 10% FBS, IC_50_ 325 μM). However, at 4 days, the cells showed sensitization to DT11 correlating with FBS level (3% FBS, IC_50_ 442 μM; 10% FBS, IC_50_ 332 μM). For DT15, in the presence of 10% FBS, the compound did not produce effects (Figure 7A, rightmost panel), while in 3% FBS (Figure 7A, second-rightmost) it demonstrated dose-dependent inhibition with comparable potency to DT11 (2 days, IC_50_ 579 μM; 3 days, IC_50_ 510 μM). In low-serum conditions at the 2-day time point, DT15 produced a small increase in viability at a 150 μM concentration.

**Figure 7:**
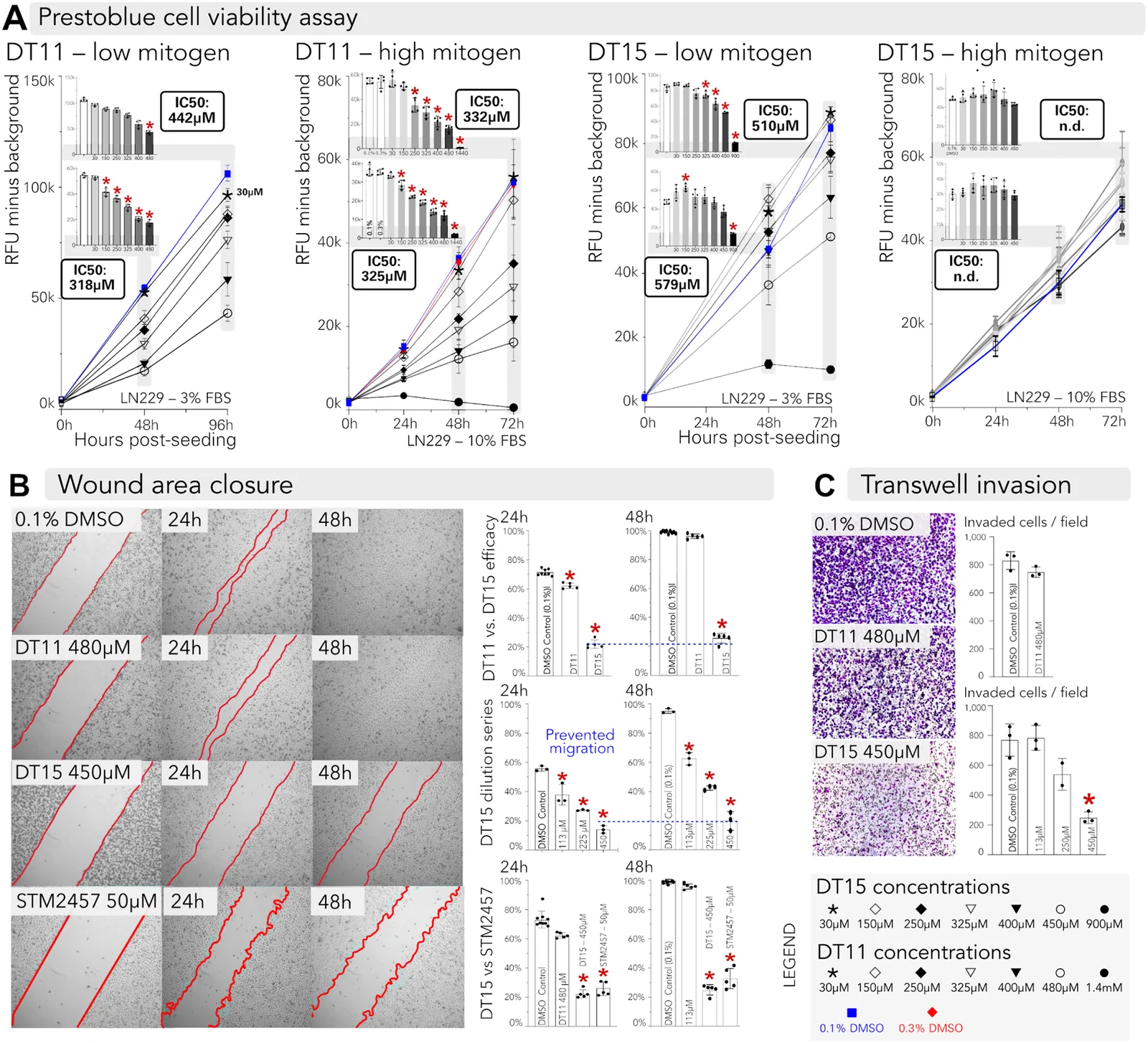
DT11 and DT15 compound effects on LN229 glioblastoma viability, scratch wound closure, and invasion. (A, left) DT11 shows dose-dependent inhibition of LN229 glioblastoma cell viability over 96-h incubation, where the IC_50_ in the 3% FBS assay (“low mitogen”) measures 318 μM at 2 days and 442 μM at 4 days. In the 10% FBS assay (“high mitogen”) over 3 day incubation, the IC_50_ measures 325 μM at 2 days and 332 μM at 3 days. (A, right) DT15 shows dose-dependent inhibition of LN229 glioblastoma cell viability over 3 day incubation, where the IC_50_ in the 3% FBS assay (“low mitogen”) measures 579 μM at 2 days and 510 μM at 3 days. Interestingly, in the 10% FBS assay (“high mitogen”), the DT15 effects on viability are lost. Data are from a representative experiment of *n* ≥ 3 separate experiments with four technical replicates. Data are presented as mean ± SD. (B) Scratch wound assay unveils DT15 to effectively prevent the closure, while DT11 shows no response. STM2457, an inhibitor of m^6^A METTL3 writer complex shown broadly to diminish m^6^A in cells,^31^ also prevented, like DT15, the LN229 cells’ scratch wound closure. DT11 and DT15 were evaluated with five technical replicates, and the 0.1% DMSO control with nine, in one experiment. Data are presented as mean ± SD. Red lines drawn for illustration. (C) DT11, at 480 μM, did not significantly inhibit LN229 cell invasion through Matrigel-coated inserts. DT15 at 450 μM resulted in 75% inhibition. Data are from three experiments and are presented as mean ± SD. RFU, relative fluorescence units of the sample after deduction of background (PrestoBlue control) fluorescence. Day 0 measurement was carried out immediately after seeding. Statistical significance was tested using one-way ANOVA with Dunnett’s multiple comparisons. *p < 0.04.

Beyond anatomical aspects, the first challenge of GBM surgical management is the optimally efficient demarcation of its aggressive infiltrative growth from brain parenchyma. Since this often fails, the clone nearly invariably relapses.^24^ Thus, we proceeded to evaluate the compounds’ effects on LN229 cells’ scratch wound closure and invasiveness. As shown in Figure 7B, only DT15 could block the closure of the scratch wound fully, while DT11 could not deliver any such effect. We considered this distinctive difference,to be mediated by DT15’s potency to inhibit m^6^A binding of all YTHDF paralogs in cells, and therefore reasoned that global downregulation of m^6^As should deliver a similar effect. For this, we administered STM2457, which has been shown to potently inhibit the catalytic subunit of m^6^A writer complex (MTC), METLL3, and thereby suppress overall m^6^A abundances in various cells.^31^ In accordance with our hypothesis, treatment of LN229 cells with STM2457 effectively prevented the scratch wound closure (Figure 7B, lowermost row). In Transwell invasion assays, DT15 prevented LN229 cell invasiveness at the same 450 μM dose as above, while DT11 had no such effect (Figure 7C). Together, the data showed that DT11 reduced cell viability, while DT15, targeting YTHDFs more broadly, had antiproliferative, perhaps antimigratory, and anti-infiltrative effects on glioblastoma cells in culture.

### Coadministration of DT11 with Temozolomide and DT15 with STM2457 Increases Efficacy

Piqued by the distinct differences in glioblastoma LN229 cell scratch wound closure distinctly linking with the m^6^A binding inhibition’s YTHDF2 selectivity or nonselectivity (Figure 7), we reasoned that, since YTHDF2 contributes to the development of TMZ resistance^32^ and, given the mechanism of DT11 is YTHDF2 m^6^A binding inhibition, it should produce a relevant additive effect when coadministered with the alkylating agent TMZ. Promisingly, at 6 days of LN229 incubation, when the TMZ’s effect on viability emerged relative to the control, 50% viability inhibition for TMZ and DT11 was reached at 200 μM and 480 μM, respectively (Figure 8A). Further, according to the hypothesis, the compounds’ coadministration at their comparable viability-inhibiting doses revealed a relevant additive effect (∼75% viability inhibition). Further, at the comparable doses for the observed phenotypes for scratch wound closure inhibition by DT15 (450 μM) and STM2457 (50 μM; Figure 7B), the compounds delivered an identical ∼40% LN229 cell viability inhibition (Figure 8B). DT15 and STM2457 coadministration revealed a relevant additive inhibition (∼70%).

**Figure 8:**
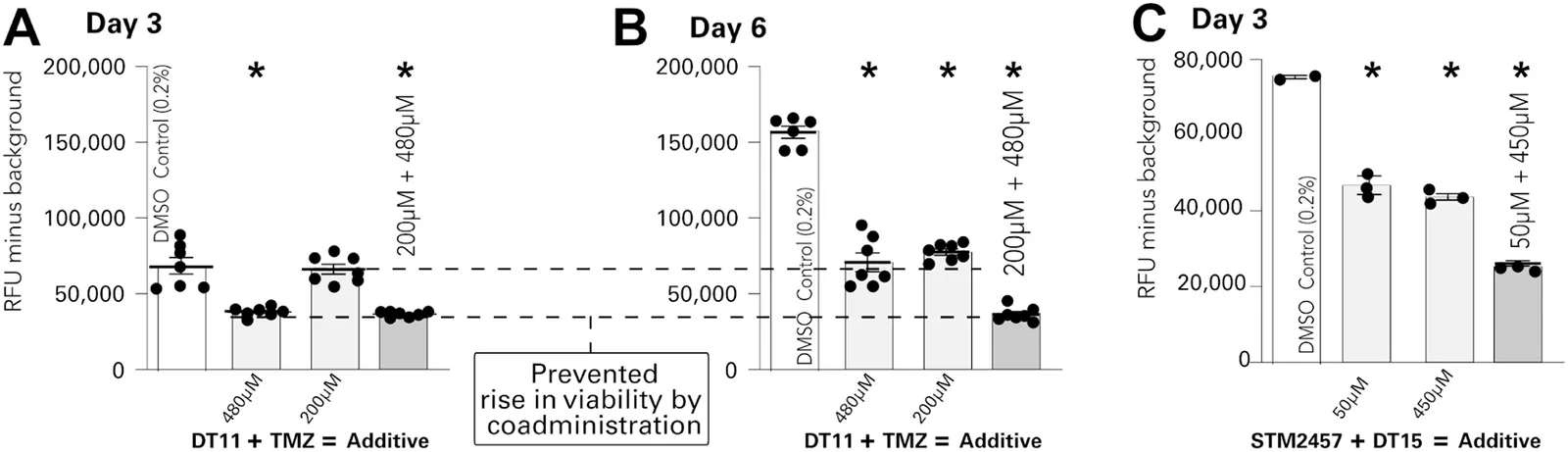
DT11, coadministered with Temozolomide, produces more pronounced viability inhibition than each alone, while DT15 acts analogously with STM2457, an inhibitor of m^6^A writer METTL3. (A, B) The head-to-head comparison of DT11 and TMZ for glioblastoma LN229 PrestoBlue viability signals at 3 and 6 days postincubation revealed comparable potencies at 6 days, which is the time point at which TMZ effects had emerged and plateaued. The DT11 efficacy was comparable at comparable doses to monotherapies, but was observable already at 3 days postincubation, whereas their coadministration showed relevant inhibitory synergy at 6 days (from 50% inhibition rates as monotherapies to a ∼75% inhibition as a dual therapy). (C) The coadministration of DT15 and STM2457 revealed an additive inhibition (∼70%). For (A), results contain two separate experiments in 10% FBS formats with data being presented as mean ± SEM (*n* = 6–7). For (B), results represent one experiment in 3% FBS formats with data being presented as mean ± SEM (*n* = 3). The difference in FBS was due to DT15 FBS-dependent effect on mitotic rate (Figure 7A). RFU, relative fluorescence units of the sample after deduction of background (PrestoBlue control) fluorescence. Statistical significance was assessed by one-way ANOVA with Dunnett’s multiple comparisons test. *p < 0.04.

### DT11 Compound Derivations Identify a 10-Fold More Potent DT11–1 for Inhibiting m^6^A Binding by the YTH-Domains

Encouraged by results with DT11 and DT15 in LN229 viability inhibition, and with the reported redundancy of YTHDF paralogs in neural systems in mind,^6,33^ we specifically selected to explore further the compound derivations for the YTHDF2-selective DT11. We forwarded to m^6^A binding inhibition assay in solution by Alphascreen with eight compound derivations of DT11, which were selected based on their commercial availability. Of these, to our knowledge, six have remained hitherto unpublished, while Invernizzi et al.,^15^ independent of this work, published the DT11-2 compound (as Compound 14) as a pan-paralog inhibitor for m^6^A binding of YTHDF1–3 YTH-domains in an HTRF assay (IC_50_ 18 μM) and *K*_d_ 3.8 μM in a fluorescence polarization assay. Of interest, the authors point-screened DT11 (as Compound S78) against YTHDF2 m^6^A binding by HTRF (IC_50_ 38 μM), yet they did not test selectivity for either YTHDF1 or YTHDF3, but found DT11 to have no effect against the uclear YTHDC2 YTH-domain (which shares 30% sequence homology with the YTHDF YTH-domains).^6^ The authors similarly also point-screened DT11-3 (as Compound S72) to find IC_50_ of 14 μM for YTHDF2. Our results for these compounds, DT11-2 (30% and 80% m^6^A inhibition at 10 μM and 100 μM, respectively) and DT11-3 (50% and 85% m^6^A inhibition at 10 μM and 100 μM, respectively), as shown in Figure 9A–B, corroborated well with these published results by Invernizzi and colleagues.

**Figure 9:**
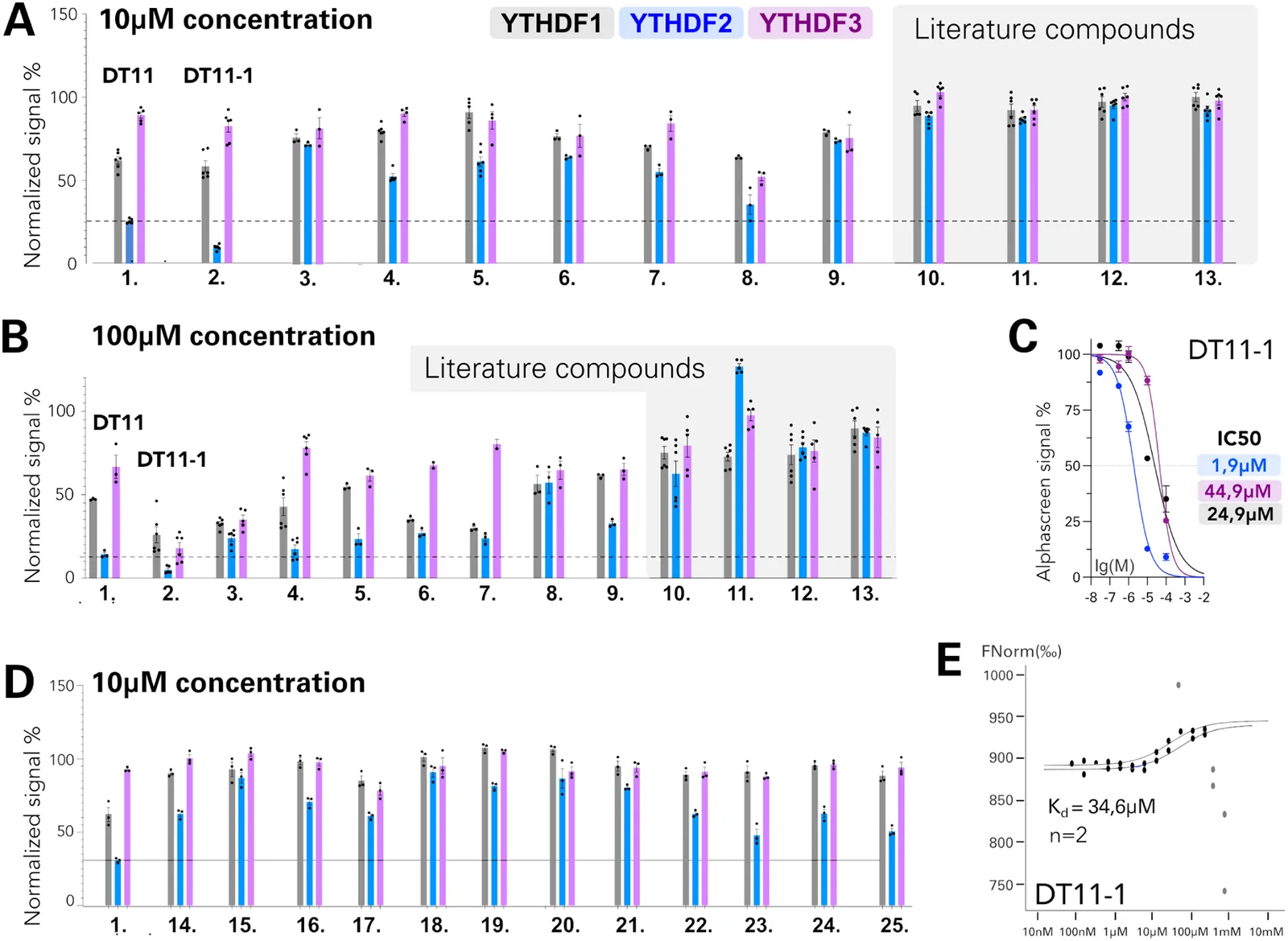
DT11 derivative compound screening identifies the 10-fold more potent DT11-1 with superior performance, along DT11, over the reported leading YTHDF2 YTH-domain-selective inhibitors. DT11 (No. 1) and its close structural derivatives (DT11-1 to DT11-8, Nos. 1–9) were screened for potency at (A) 10 μM and (B) 100 μM for inhibition of m^6^A binding to RNA against all human YTHDF1–3 paralogs’ YTH-domains. The structure of DT11-1 (No. 2) demonstrated YTHDF2 selectivity and (C) with near 10-fold potency in inhibiting m^6^A binding to RNA (IC_50_ = 1.9 μM for YTHDF2) over the DT11. Head-to-head comparisons of leading published YTHDF2-targeting selective inhibitors DC-Y13–27 (No. 11),^14^ CK-75 (No. 12),^34^ DF-A7 (No. 13),^35^ and CCI-38 (No. 10)^36^ revealed considerably weaker potencies in the inhibition of binding the m^6^A on RNA. (D) Further DT11 derivative screening further establishes the superiority of DT11 and DT11-1 for the m^6^A binding inhibition. (E) MST for DT11-1 against YTHDF2 revealed *K*_d_ = 34.6 μM. (A–B) Results from two separate experiments. data are presented as mean ± SEM (*n* = 3–6). (C) Results from one experiment. Data are presented as mean ± SEM (*n* = 3). (D) Results from one experiment. Data are presented as mean ± SEM (*n* = 3). (E) Results from two experiments. Data are presented as mean ± SEM.

Notably, however, by exploring little modifications of the DT11 chemical structure, that is, adding a methyl N-substitution at positions 5 and 3 (i.e., both sides of the carbonyl group), the newly established compound, termed as DT11-1, inhibited YTHDF2 m^6^A binding with 10-fold potency but without a compromise in selectivity. The IC_50_ values for DT11-1 against m^6^A binding in RNA by YTHDF1, YTHDF2, and YTHDF3 were 24.9 μM, 1.9 μM, and 44.9 μM, respectively (Figure 9C). Encouraged, we turned to test still 11 DT11-scaffold-based compounds, including DT11-20 with ethyl, instead of DT11-1’s methyl, N-substitution at position 5 next to the carbonyl group, and observed no better potency or efficacy of inhibition reached by these derivatives (Figure 9D), which were also selected based on their availability. Thus, we focused on DT11-1 and validated its interaction with YTHDF2 by MST, revealing it with *K*_d_ 34.6 μM (Figure 9E). Notable deflections in the signal were seen in the high micromolar to low millimolar range. All the compound structures of DT11 derivatives and the head-to-head tested literature control compounds, those discussed below, are listed in Table 1.

**Table 1:**
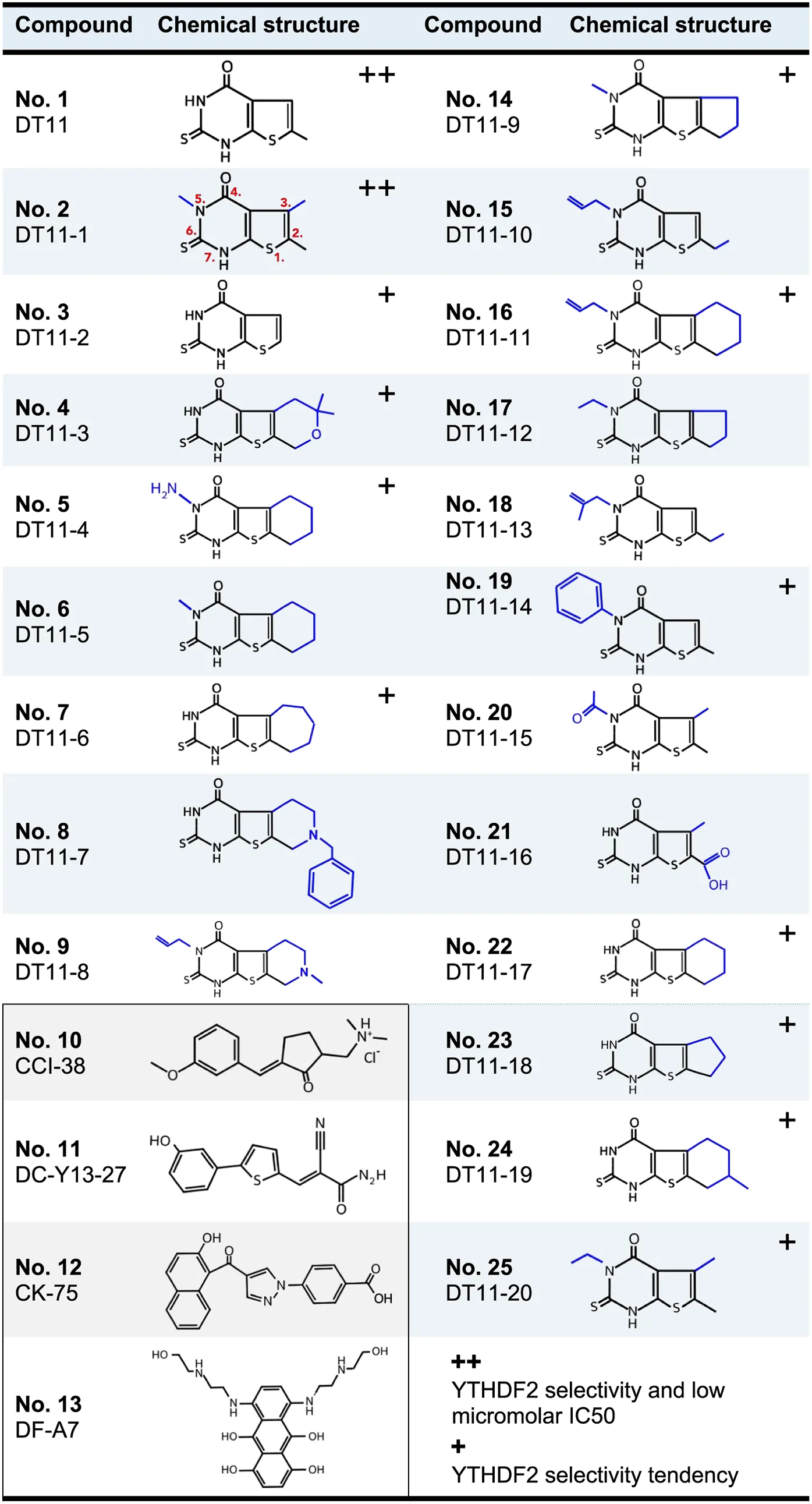
DT11 Derivative Compounds’ Chemical Structures

### Examination with YTHDF2-Selective Literature Compounds Reveals DT11 and DT11-1 Superiority in Inhibiting m^6^A Binding of Human YTHDF YTH-Domains

Encouraged by this finding, the potencies of DT11 and DT11-1 in m^6^A binding inhibition were head-to-head compared with the leading published YTHDF2-targeting selective inhibitors DC-Y13–27,^14^ CK-75,^34^ DF-A7,^35^ and CCI-38.^36^ Notably, as illustrated in Figure 9A,B, based on two repeated experiments with six pooled technical replicates, the only compound that showed YTHDF2-selective inhibition of m^6^A binding in solution was CCI-38, and it exhibited lower potency (15% and 40% m^6^A binding inhibition at 10 μM and 100 μM, respectively) than *K*_d_ of 10.1 μM from MST by authors.^36^ The results posit the here-identified DT11 and DT11-1 superior.

### DT11-1 10-Fold Potency over DT11 in Inhibiting m^6^A Binding Replicates in the LN229 Glioblastoma Cell Viability Assay

With the insight on the superior potencies of DT11 and DT11-1 in m^6^A binding inhibition over reported YTHDF2-selective interactor compounds (Figure 9A,B), we asked whether this potency increase (from DT11 to DT11-1) would be reflected in the viability-inhibition potency of the LN229 glioblastoma cells as well. In support of the hypothesis that the viability inhibition of these YTHDF2-dependent cells^25^ would be mediated by the here-designed selective m^6^A binding inhibition of YTHDF2, a similar reduction in viability signal required only ∼45 μM of DT11-1, while, in the head-to-head comparison, DT11 required ∼480 μM (Figure 10A). This result—the full correlation of YTHDF2 m^6^A binding inhibition potencies to the LN229 cells’ viability-inhibition potencies—together with DT11’s relevant additive inhibition with TMZ (Figure 8A,B), supports the idea that the mechanism of action could be YTHDF2-selective m^6^A binding inhibition in cells.

**Figure 10:**
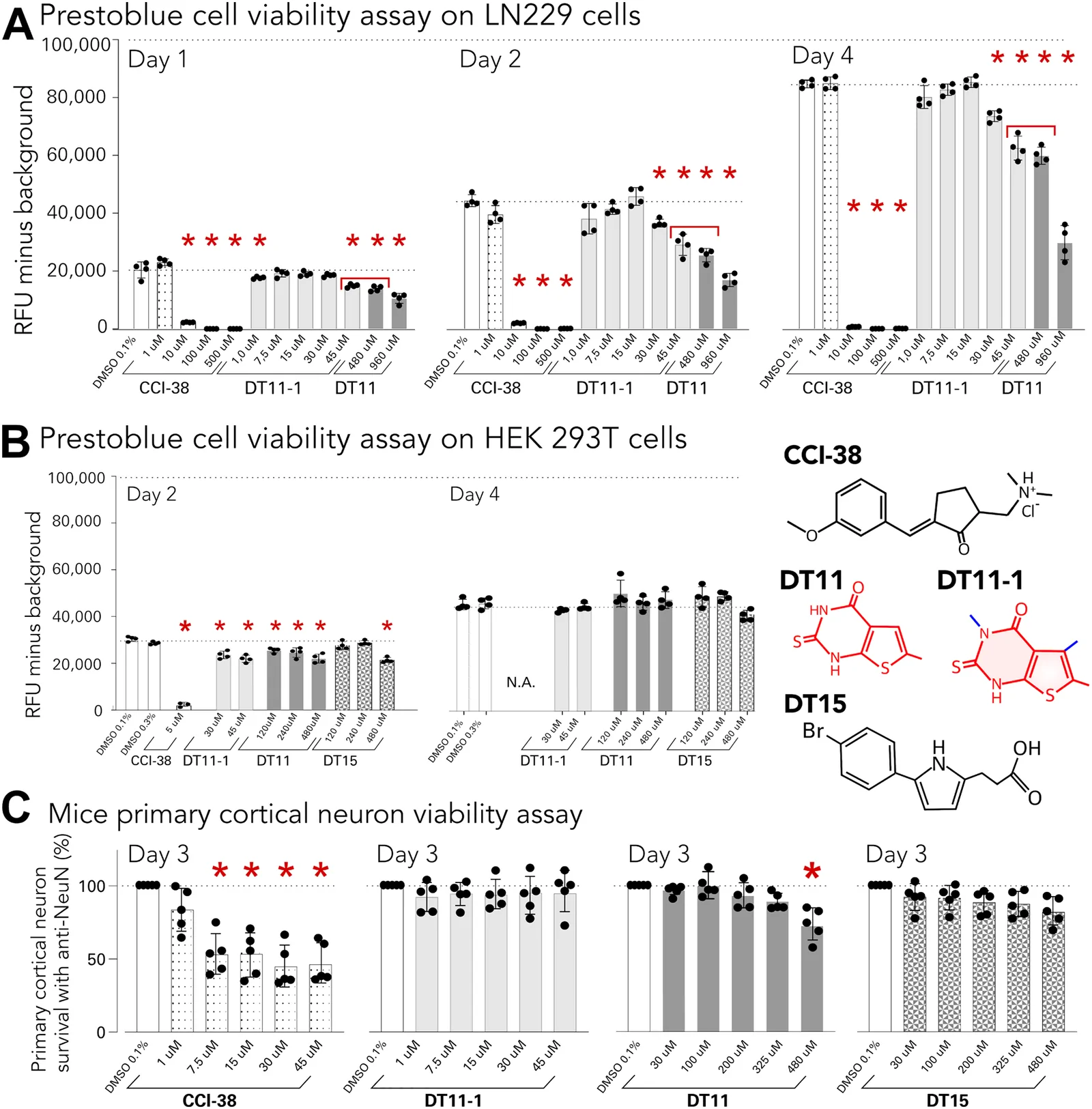
LN229 evaluation of DT11-related chemistry and toxicity profiles in mitotic and postmitotic cells. (A) A head-to-head evaluation of the LN229 glioblastoma cell line viability inhibition potencies for DT11, DT11-1, and CCI-38. Remarkably, minute methyl-group additions to the DT11 compound, yielding DT11-1, established in full correlation the 10-fold potency, which was first observed for YTHDF2 m^6^A binding inhibition (without compromise in selectivity), here at the cellular level. Indeed, to reach the equivalent inhibition rate reached by DT11 at 480 μM, only 45 μM of DT11-1 were required. Notably, CCI-38, among the leading published YTHDF2-selective inhibitors (with no test of m^6^A binding inhibition *per se*), killed all LN229 cells already at 10 μM, and showed no effect at 1 μM, evidencing of a narrow efficacy range at a discordantly low dose relative to the observed YTHDF2 m^6^A binding inhibition (Figure 9). (B) A head-to-head evaluation of the HEK293T viability inhibition potencies for DT15, DT11, DT11-1, and CCI-38. Notably, at day 4, when inhibition for LN229 cells was seen, no effects were seen for DT11, DT11-1, and DT15 in these benign cells, while CCI-38 showed full inhibition and killed the cells, suggesting toxicity. (C) Primary cortical neuron culture *ex vivo* revealed, at 3 days of incubation, CCI-38’s toxicity at a low micromolar dose, while DT11 and DT15 showed minimal to nonexistent toxicity at the inhibitory LN229 doses. The more potent DT11-1 had no toxicity at the comparable inhibitory dose for LN229 cells as DT11. For panel (A), data are from two separate experiments with four technical replicates each and are presented as mean ± SD. For panel (B), data are from two separate experiments with six technical replicates each and are presented as mean ± SD. For panel (C), data are from five separate experiments with one technical replicate each and are presented as mean ± SD. RFU, relative fluorescence units of the sample after deduction of background (PrestoBlue control) fluorescence. Statistical significance was assessed by one-way ANOVA with Dunnett’s multiple comparisons. *p < 0.04.

### DT11, DT11-1, and DT15 Demonstrate No Viability Inhibition of HEK293 Cells, While the Leading Reported YTHDF2-Selective Inhibitor CCI-38 Kills the Cells

Encouraged by the results in the solution experiments and the cellular effects on viability, invasion, and migration, together with a full correlation of potencies from m^6^A binding inhibition to LN229 viability inhibition, from the DT11 design toward DT11-1, comparable efficacy with the gold standard TMZ, and DT11’s additive inhibition with TMZ, we turned to evaluate the compounds, along with CCI-38, in benign proliferating HEK293T cells. At 2 days, with the doses where DT15, DT11, and DT11-1 demonstrated notable viability inhibition in LN229 cells, the compounds had zero efficacy in HEK293T cells (Figure 10B). This suggests the compounds’ have emerging selectivity for malignant cells. Notably, after 1 day of incubation, CCI-38, at 5 μM, a low dose relative to the reported interaction with the YTHDF2 YTH-domain (*K*_D_ 10.1 μM),^36^ fully inhibited cell viability and, upon inspection, had killed the cells. This result suggested that this compound, recently proposed as a promising agent for B-cell malignancies *in vitro* and *in vivo*,^36^ has toxic effects at relatively low doses. We further examined these thoughts in primary cortical neurons.

### DT11, DT11-1, and DT15 Demonstrate Little to No Toxicity in Primary Cortical Neurons, While the CCI-38 Is Toxic at the Dose of LN229 Glioblastoma Effects

Finally, we conducted a head-to-head evaluation of the toxicities of DT11, DT15, DT11-1, and CCI-38 in native mice primary cortical neurons *ex vivo*. Concordant with the finding that CCI-38 did not potently inhibit m^6^A binding even at 100 μM with purified YTHDFs and m^6^A-RNA (Figure 9B), yet demonstrated a full loss of LN229 cell viability at 10 μM (Figure 10A), the compound, already at 1 μM with a three day incubation showed ∼25% toxicity in the primary neurons, which plateaued and reached a significant ∼50–60% kill rate at 7.5 μM (Figure 10C). Given that the IC_50_ values for LN229 cell viability inhibition and primary cortical neuron toxicity coalesced and were much lower than CCI-38’s m^6^A binding inhibition potency, together with a *K*_d_ over 4 mM for the YTHDF2 YTH-domain in MST (*n* = 3, data not shown), we consider it uncertain whether the compound inhibits YTHDF2 binding m^6^A on RNA and rather suspect it acts via a distinct mechanism toxic to neurons. This notion is stressed further by finding that DT11 induced only little toxicity at the verified IC_50_ for YTHDF2 m^6^A binding (480 μM), which was fully lost for DT11-1 at an equivalent concentration (45 μM), and DT15 showed no statistical effects (Figure 10C). Together, the data suggest DT11, DT11-1, and DT15 as quite promising compound scaffolds for pharmaceuticals.

## Discussion

We report here the discovery of compounds with and without selectivity for inhibiting the m^6^A binding of YTHDF paralogs in RNAs. From our *in silico* high-throughput screening, the *in vitro* assay validation found DT11 to deliver a marked—10–100-fold—differences in its IC_50_ for the YTH-domain of YTHDF2 m^6^A binding over that of YTHDF1 and YTHDF3, respectively. We verified the selectivity with HTRF, assessed direct binding with MST, and validated it with NMR. Along with the unselective inhibitor, DT15, we demonstrate DT11’s efficacy to suppress viability of the LN229 GBM cell line, which is shown to be YTHDF2-dependent and correlates with its prognosis.^25^ Further, DT11 coadministration with TMZ, the gold-standard GBM chemotherapeutic,^37^ was seen here to award an additive effect on viability inhibition. By dosing LN229 cells with DT11 or DT15—and so exposing no effect or blocked invasion and scratch wound closure, respectively—we elucidated distinct phenotypes to link with distinct modes of inhibition, YTHDF2 selectivity or nonselectivity across the paralogs. We showed the compounds’ little to nonexistent toxicity for primary cortical neurons and their lack of activity on the viability of benign proliferative HEK293T cells. Here, however, the CCI-38 compound was comparably toxic to these cells as to cortical neurons. We described DT11 derivatives to find DT11-1 with retained selectivity, 10-fold enhanced potency both in the purified system and in GBM cells, and loss of the little neuronal toxicity found with DT11. Thus, by the rationally designed antagonism of recognition of m^6^A in RNA, our results support the recent synthesis that human YTHDF paralogs do differ even at their conserved YTH-domains, a focus of recent discussion on the extent of unity of their cellular functions,^6–10^ apt for new action pharmacodynamics with promising therapeutic prospects.^6^

To the best of our knowledge, this is the first work on compounds targeting YTHDF paralogs, and the epitranscriptome overall, against GBM. The rationale to design novel compounds for GBM is supported by the fact that its dismal prognosis is not improving, despite the advent of cancer vaccines, Gamma Knife procedures , and neoadjuvants.^38^ Second, the invasiveness and proliferation of GBM have been shown to depend on YTHDF2, with a link to its N-terminal tail phosphorylation.^25^ Third, increasing YTHDF2 has been recently found alongside increasing GBM malignant grade.^25^ Fourth, YTHDF2 participates in the development of TMZ resistance.^32^ Thus, our data on the DT11-TMZ additive effects support its wider study, like those overcoming resistance. The molecular dynamics corroborating the solution assays, the full correlation of DT11 and DT11-1 m^6^A binding inhibition potencies with LN229 cell viability inhibition potencies, and phenocopied DT15’s scratch wound closure effects by STM2457, altogether support the idea that DT11 and DT15 inhibit YTH-domain m^6^A binding by YTHDF2 and YTHDF1–3, respectively. The fact that DT15 could block the GBM cells’ scratch wound closure and invasiveness fully, but DT11 did not have any such activity, suggests these functions involve a YTH-domain-linked cross-paralog redundancy. Considering the first challenge of GBM management against its aggressive infiltrative growth to parenchyma,^24^ and keeping the increased m^6^A within the invasive rim of GBM in mind,^26^ this DT15-linked block of cell invasion warrants refinement of potency, despite the absence of cortical neuron toxicity even at current micromolar doses.

Several aspects of the data warrant notes. The causes of the additive effects of DT15 and STM2457 can be several. First, m^6^A is written by also methyltransferases beyond MTC (where the METTL3 is the active subunit), including METTL16 and METTL7A,^6^ which are not targeted by STM2457, and likely start to compensate upon MTC’s incapacitation.^31^ Of note, a recent abstract showed a similar STM2457 effect to reduce GBM cell invasion.^39^ Second, cells contain a plethora of other, whether direct or indirect, m^6^A binding reader proteins and families beyond the YTHDF family, which are not expected to be targeted by DT15.^6^ Third, both compounds may formally act via off targets. The additive effect of DT11 and TMZ likely arises from distinct mechanisms; TMZ acts by alkylation affecting the epigenome and transcription, while, as designed here, DT11 acts on the epitranscriptome and so translation.

For the *in vitro* protein assays, several facts also emerge. Considering we could not measure the perhaps expected potent inhibition of m^6^A binding to YTHDF1–3 by the CCI-38 compound, it is noteworthy that the constructs, buffer, and detection methods were different here. Indeed, the original paper, describing YTHDF2-selective activities of CCI-38, did not test for the m^6^A binding inhibition, but used drug affinity responsive target stability (DARTS) detecting interactions.^36^ Given its Michael acceptor moiety’s is prone to interact with cysteines, off-target testing appears warranted. An analogous situation concerns the report of the DF-A7 compound, which was denoted as a YTHDF2 specific degrader at IC_50_ ∼ 50 nM.^35^ Our data revealed a mere ∼10% inhibition of m^6^A binding across all paralogs at 100 nM of DF-A7. Considering these data, which, to the best of our knowledge, are the first on the compound’s interaction with YTHDF paralogs, we consider the reported YTHDF2 “specific” degradation, and neither YTHDF1 nor YTHDF3,^35^ to be a likely result of another target in a pathway, stressing examination of direct interactions. Further, it remains interesting that in our setup, where the native signal-inert m^6^A-RNA displaced the signal-potent m^6^A-RNA at the nanomolar range, neither DC-Y13–27 ^14^ nor CK-75 ^34^ could approach their IC_50_ values reported in their original reports. However, in support of YTHDF2-selective effects of DC-Y13–27, despite in opposite to inhibition, we measured here a tendency for YTHDF2-selective m^6^A binding activation.

Also, the raw m^6^A binding signals varied across paralogs. This likely reflects the fact that the paralogs possess, as recently reviewed,^6^ amino acid differences in their YTH-domains. Across the paralogs, the domain folds into an α/β fold with three α-helices and six β-strands, of which the aromatic cage, binding the m^6^A, comprises partially of two α-helices and four β-sheets with three key tryptophans.^11^ Further, the pocket’s outer wall is formed by an m^6^A-recognition loop, which remains in a primary peptide configuration.^6^ Despite the similarity of YTH-domains among the paralogs, there remain nonconserved amino acids in and around the loop. Mutagenesis has shown that altering the loop leads to altered m^6^A binding,^6^ and molecular dynamics reveal paralog-selective effects.^13^ Recently, Cazzanelli et al. published an apo crystal structure of YTHDF1 to show its distinct configuration when lacking m^6^A-RNA, thus proposing a model in which the cage exists in an equilibrium driven by the loop’s dynamics.^40^ These functions of the loop can also underlie the observed selectivity for DT11, especially since several residues from it were found to interact with DT11 in the simulations. This biology can also underlie, through solution details, the distinct results we found for DC-Y13–27 ^14^ and CK-75.^34^ Analogously, differences between the MST buffer and the AlphaScreen buffer can thus partake in some of the differences between DT11’s and DT15’s IC_50_ and *K*_d_ values.

We consider key strengths of our study in the dedicated design of novel-acting compounds, those disrupting the YTHDF YTH-domain m^6^A binding to RNAs selectively and nonselectively across these paralogs, and their first-of-its-kind GBM effects with the desirable benign-to-malignant cell effects. Another strength lies in the systematic use of the natural m^6^A-ligand-inhibition-controlled m^6^A binding assay against all three YTHDF YTH domains, head-to-head, as well as in the systematic comparison of the here-reported novel compounds with the established literature compounds for, to our best knowledge, the first time. Future work should address pharmacokinetics, including penetration of the blood–brain barrier, extend the cell lines, evaluate *in vivo* efficacies, and still refine potencies, as fueled by the compounds here, for example, with the use of quantitative structure–activity relationship (QSAR) methods. Keeping the desired cortical neuron tolerance in mind, the compounds could be seen also worth testing in various neurodegeneration models, for example, alongside the recently reported m^6^A eraser FTO inhibitors^41^ and MTC activators,^42^ the emerging neuroprotective compounds acting comparably to, or even exceeding, the gold standard glial cell line-derived neurotrophic factor (GDNF).

## Conclusion

We discovered here DT11, and its derivative DT11-1, with marked selective inhibitory potencies for the YTHDF2 YTH-domain binding to m^6^A in RNA, together with the nonselective compound DT15. Based on relevant cellular readouts with distinct phenotypes linked to the designed modes of inhibition—the YTHDF2-selectivity or nonselectivity at the conserved m^6^A binding aromatic cage—they emerge as a promising foundation to refine the here-reported line of pharmaceutical chemistries for epitranscriptomic pharmacology to harness the potential vested in the epitranscriptome.

## Materials and Methods

### In Silico Screening and Structural Optimization of YTHDF Binding Compounds

Computational docking was employed to identify compounds with high affinity for binding to the RNA m6A reader proteins YTHDF1, YTHDF2, and YTHDF3. The 3D structures of these m6A reader proteins were selected to describe potential target binding sites for small-molecule inhibitors. The crystal structures of YTHDF1 (PDB: 4RCJ),^11^ YTHDF2 (PDB: 4RDN),^12^ and YTHDF3 (PDB: 6ZOT)^13^ were applied for subsequent modeling. Prior to molecular docking, the raw crystal structures of all target proteins were refined using Schrödinger’s Protein Preparation Wizard.^43^ In order to identify potential ligands binding to YTHDF1, YTHDF2, or YTHDF3, high-throughput virtual screening of the FIMM database was performed using the Glide Virtual Screening workflow within the Schrödinger LLC suite.^28,29^ The binding interfaces between the cocrystallized ligand and receptor for each target protein were delineated using the Schrödinger Glide Grid Generation procedure.^28^ The precise coordinates of these identified active sites were then employed in molecular docking analyses. Ligand structures were optimized via the density functional theory (DFT) B3LYP method^44^ with the 6-31G basis set.

Ligands exhibiting the highest docking efficiency (DE) values were selected for subsequent experimental validation. A total of 36 ligands were identified for YTHDF1, 35 for YTHDF2, and 31 for YTHDF3.

DEs were calculated using the following equation:

1 $$DE=-\frac{\Delta G_{\mathrm{dock}}}{N}$$

where Δ*G*_dock_ represents the docking free energy, and *N* is the number of non-hydrogen (“heavy”) atoms in the small molecule.

Two compounds, DT4 and DT13, that demonstrated activity in *in vitro* experiments were subjected to further structural optimization to identify new ligands with enhanced activity toward YTHDF family proteins. The new compounds, featuring different functional groups of small-molecule ligands, were prepared for docking using the Schrödinger Maestro LigPrep procedure^45^ and docked to the active site of each of the studied target proteins using the Schrödinger’s Glide procedure.^28,30^ The docking parameters were set to their default values. Ligands exhibiting the highest DE values were selected for subsequent experimental validation.

Molecular dynamics simulations were performed using the Desmond simulation package from Schrödinger LLC.^46^ All simulations were conducted under the NPT ensemble at a temperature of 300 K and a pressure of 1 bar. For each protein (YTHDF1, YTHDF2, and YTHDF3), five independent 10 ns runs were performed, with a relaxation time of 1 ps, focusing on the interactions between the ligands DT11 and DT15 and the proteins. The OPLS_2005 force field parameters were applied throughout.^47^ Ligand–protein behavior and interactions were analyzed using the Simulation Interaction Diagram tool integrated into the Desmond Molecular Dynamics package. The stability of the simulations was assessed by tracking the RMSD of ligand and protein atomic positions over time.

### In Vitro Screening of Candidate Compounds for YTHDF Binding to m^6^A in RNA

We used a proximity- and phthalocyanine-based singlet oxygen-producing AlphaScreen method to assess the YTHDF binding to m^6^A modified RNA oligonucleotide (m^6^A-RNA) and to conduct consequent inhibitor screening. The AlphaScreen anti-FLAG detection kits were used (PerkinElmer, Waltham, MA, USA). The displacement assays were performed with PerkinElmer polystyrene 1/2 Area AlphaPlate-96 light-gray plates (catalog ref 6002350, Waltham, MA, USA). The RNA oligonucleotides were purchased from Immuno Diagnostic Ltd. (Hämeenlinna, Finland) and produced by Metabion International AG (Planegg, Germany). The sequences of all the 27-nucleotide-spanning oligonucleotides are listed in Table 2. The assays were performed in a HEPES-based buffer optimized for RNA-binding proteins to minimize aggregation (HEPES 30 mM, pH 7.5, KCl 100 mM, NaCl 40 mM, ammonium acetate 10 mM, guanidinium hydrochloride 10 mM, MgCl_2_ 2 mM, EDTA 0.5 mM, DMSO 3%, and NP-40 0.01%), as earlier described.^48^ For the recombinant YTHDF proteins, FLAG-tagged recombinant human YTHDF1 (380-533, Catalog No: 81102; Active Motif, California, USA), YTHDF2 (401-554, Catalog No: 81103; Active Motif, California, USA), and YTHDF3 (407-560, Catalog No: 81104; Active Motif, California, USA) were utilized. Upon arrival, the RNA probes were dissolved in RNase-free water (Invitrogen Ultrapure DNase/RNase-free distilled water, catalog ref 10977035), while the proteins were stored in the HEPES buffer upon aliquoting. The RNAs were stored at −70 °C, while proteins were stored in liquid nitrogen (−196 °C).

**Table 2:** RNA Oligonucleotides Used in the Study

probe name	sequence
B-m^6^A-RNA	5′-Biotin-UAC ACU CGA UCU GG(**N6-Me-rA**) CUA AAG CUG CUC-Biotin-3′
B-A-RNA	5′-Biotin-UAC ACU CGA UCU GG**A** CUA AAG CUG CUC-Biotin-3′
Comp-m^6^A-RNA	UAC ACU CGA UCU GG(**N6-Me-rA**) CUA AAG CUG CUC

To assess the binding efficacies and m^6^A selectivities of recombinant YTHDF1, YTHDF2, and YTHDF3, proteins were incubated with m^6^A-methylated and nonmethylated biotinylated RNAs (i.e., B-m^6^A-RNA and B-A-RNA, respectively, Table 2). First, recombinant YTHDFs, biotinylated RNAs, and anti-FLAG-conjugated AlphaScreen acceptor beads, 10 μL of each, were mixed in the wells and incubated for 30 min. Next, 10 μL of streptavidin AlphaScreen donor beads were added to the wells, followed by another 30 min incubation. Incubations were performed at room temperature, protected from light, with gentle shaking. Plate readings were done with an EnVision 2105 multimode plate reader (PerkinElmer, Waltham, MA, USA) with an AlphaScreen 570 emission filter, with 180 ms for excitation and 370 ms for emission. After initial screening for optimal binding efficacy and selectivity for m^6^A modification, displacement and inhibitor screening assays for YTHDF1, YTHDF2, and YTHDF3 were conducted.

Prior to inhibitor screening, to verify and scope the signal inhibition potential of the assay, a displacement competition test was performed for all YTHDF1, YTHDF2, and YTHDF3 proteins utilizing an m^6^A-methylated nontagged RNA probe (i.e., Comp-m^6^A-RNA, Table 2) in a dilution series and in technical triplicates. The assay was done by adding 3 μL of Comp-m^6^A-RNA to the protein, B-m^6^A-RNA, and acceptor beads for a 30 min incubation prior to adding the donor beads.

To perform the inhibitor screening assays, wells in 384-well plates were coated with 1 μL of the examined potential inhibitor compounds in dilutions. For assessing inhibition, 10 μL of supermix, containing AlphaScreen beads, B-m^6^A-RNA, and YTHDF YTH-domain recombinant constructs, was dispensed into the wells by a CERTUS FLEX (Fritz Gyger AG, Gwatt, Switzerland) with the buffer-optimized 150 mbar pressure. Then, the plate was point-spun at 5000 rpm and incubated for an hour as described for the manual assays above.

### The Homogeneous Time-Resolved Fluorescence (HTRF)

A proximity-based HTRF assay using time-resolved fluorescence resonance energy transfer (TR-FRET) was used to validate DT11’s capability to selectively inhibit YTHDF2’s ability to bind double-biotinylated m^6^A-modified RNA. In the assay, anti-FLAG-conjugated Terbium donor beads (#61FGBTLF) and streptavidin-coated XL665 acceptor beads (#610SAXLF) were purchased from Revvity (Waltham, USA). Upon arrival, the lyophilized product was diluted in distilled water according to the manufacturer’s manual and stored at +4 °C. Assays were done in shallow 384-well ProxiPlate plates (#6008280, Revvity).

To perform the inhibitor screening assays, designated wells of 384-well plates were coated with 1 μL of the inhibitor compound in a dilution series. For assessing signal inhibition, a displacement assay was performed, where, instead of the candidate inhibitor compound, nonbiotinylated, and so signal-inert in terms of the streptavidin–biotin interaction-dependent HTRF, identical m^6^A RNA was added to the wells to observe signal inhibition by this natural ligand of the site. Next, 10 μL of supermix containing HTRF donor and acceptor beads, m^6^A RNA oligos, and recombinant YTHDF paralog was pipetted into the wells, with careful up-and-down pipetting to assist proper solution mixing. The final well volume of 11 μL contained HTRF beads at a 1:75 dilution. For each YTHDF paralog, the recombinant YTH-domain and m^6^A RNA concentrations were selected based on my previous assay optimization results (data not shown): YTHDF1 assay: m^6^A RNA 50 nM, YTHDF1 75 nM; YTHDF2 assay: m^6^A RNA 50 nM, YTHDF2 65 nM; YTHDF3 assay: m^6^A RNA 60 nM, YTHDF3 300 nM. Briefly, optimized concentrations were determined through titration experiments in which YTHDF paralogs and m^6^A RNA were titrated against each other to identify conditions that maximized signal production and assay sensitivity.

Next, plates were sealed, mixed for 10 s on a plate shaker, spun, and incubated for 1 h on a plate shaker at room temperature (RT). After incubation, the plate was read with a PerkinElmer (Waltham, USA) EnVision 2105 multimode plate reader, with an excitation filter for terbium excitation of 340 nm and emission filters for terbium emission of 545 nm and XL665 emission of 665 nm. A 70 ms time delay between excitation and emission capture was utilized to minimize nonspecific background fluorescence. The signal at 665 nm emission was normalized to the 545 nm emission baseline.

### Microscale Thermophoresis

Microscale Thermophoresis (MST) was performed to investigate the binding of DT11 to YTHDF recombinant proteins. Briefly, recombinant YTHDF1 (#81102), YTHDF2 (#81103), and YTHDF3 (#81104) (Active Motif, Carlsbad, USA) were labeled using the His-Tag Labeling Kit RED-tris-NTA Second Generation (#MO-L018; NanoTemper, Munich, Germany) according to the manufacturer’s instructions. Both the labeled recombinant protein and DT11 were diluted in MST buffer (10 mM Na-phosphate, pH 7.4, 1 mM MgCl_2_, 3 mM KCl, 150 mM NaCl, 0.05% Tween 20). Equal volumes (5 μL) of labeled protein and DT11 were mixed in a tube and incubated for 30 min on ice. The final protein concentration was 20 nM, and DT11 was tested in a 14-point dilution. Mixture was suctioned in Monolith Premium Capillaries (#MO-K025; NanoTemper) and applied to the Monolith NT.115 (NanoTemper) device.

To investigate if DT11 can interfere with recombinant YTHDF proteins binding to m^6^A-RNA competition assays were performed. First, recombinant YTHDF proteins’ binding affinity with NB-m^6^A-RNA was assessed using the same MST protocol as for DT11. For the competition assay, labeled recombinant YTHDF proteins, diluted in MST buffer, were preincubated with DT11 (100 μM) for an hour on ice. After preincubation, equal volumes (5 μL) of preincubated recombinant protein were mixed with NB-m^6^A-RNA oligonucleotide (diluted in MST buffer) and incubated for 30 min on ice. The final protein concentration was 20 nM, and DT11 was tested in a 14-point serial dilution. The mixture was then suctioned in Monolith Premium Capillaries (#MO-K025; Nanotemper) and applied to a Monolith NT.115 (Nanotemper) device. MST data were analyzed using MO.Affinity Analysis software (Nanotemper).

### Protein Expression and Purification for NMR

The YTHDF2 (401–554) in the pGTvL1-SGC vector was expressed in standard M9 minimal medium supplemented with 1 g/L ^15^N NH4Cl (Cambridge Isotope Laboratories) and 2 g/L Glucose (Cambridge Isotope Laboratories) as the sole nitrogen and carbon sources, respectively. Cells were grown until OD_600_ was equal to 0.7, and protein expression was induced by adding 0.4 mM isopropyl-β-d-1-thiogalactopyranoside (IPTG) and incubating the culture at 18 °C, 230 rpm for 20 h. Cells were harvested by spinning at 6000 × *g* at 4 °C for 20 min. The cell pellet was resuspended in 1× PBS , spun at 10.000 × *g* at 4 °C for 10 min, and stored as a pellet until purification.

After lysis with an EmulsiFlex in 1× PBS supplemented with 5% Glycerol, 5 mM DTT, 0.2 mg DNase I (Roche), and 100 U RNase (Sigma), YTHDF2 was purified using Protino Glutathione Agarose 4B (Macherey-Nagel). Protein was eluted using the same buffer supplemented with 50 mM glutathione (Sigma), and then GST was cleaved by in-house-made Tobacco Etch Virus (TEV) protease at 4 °C for 16 h. Protein was further purified to homogeneity by size-exclusion chromatography (SEC) using a HiLoad 26/60 Superdex 75 pg column (GE Healthcare) in SEC buffer (20 mM Tris-HCl, pH 7.5, 150 mM NaCl, 2 mM DTT) on an ÄKTA pure system (GE Healthcare). The protein was concentrated using an Amicon Ultra centrifugal 10K filter device (Merck Millipore Ltd.) to the desired concentration.

### NMR Spectroscopy and ^15^N YTHDF2 Titration with DT11

All NMR spectra were measured at 25 °C, using a Bruker Avance III HD 800 NMR spectrometer, equipped with a cryogenically cooled ^1^H, ^13^C, and ^15^N TCI probehead with an actively shielded z-gradient coil. For NMR titration, 30 mM HEPES, pH 7.5, 100 mM KCl, 40 mM NaCl, 10 mM CH_3_COONH_4_, 10 mM Guanidium hydrochloride, 2 mM MgCl_2_, 0.5 mM EDTA, 3% DMSO, and 0.01% NP-40 buffer was used (1× titration buffer, Mills et al.^48^). To prepare the ^15^N-YTHDF2 sample reference for the NMR binding study, the protein stock was diluted to 100 μM using a 5× concentrated titration buffer. Then, DT11 was resuspended in 100% DMSO to a concentration of 100 mM and further diluted to 10 mM in 1× titration buffer. Aliquots of DT11 corresponding to 0.5x, 1x, and 2× were dried using a Savant SpeedVac. Protein was added to dried DT11, and ligand-binding-induced CSPs were monitored using an ^15^N-HSQC experiment at each titration point.

### Glioblastoma and HEK293T Cell Cultures

Human GBM LN229 cells were obtained from Cytion (#305043, Eppelheim, Germany). Cells were grown in high-glucose Dulbecco’s Modified Eagle Medium (DMEM; #41966029, Thermo Fisher Scientific, Waltham, USA), supplemented with 10% Fetal Bovine Serum (FBS; #A5256801, Thermo Fisher Scientific) and 1% Penicillin–Streptomycin (P/S; #15140-122, Thermo Fisher Scientific), and incubated at +37 °C in a humidified atmosphere with 5% CO_2_. HEK293T cells were obtained from Cytion (#305043, Eppelheim, Germany) and maintained as the LN229 cells.

### PrestoBlue Cell Viability Assay

Cell viability following compound exposure was assessed using the PrestoBlue High-Sensitivity reagent (#P50201, Thermo Fisher Scientific) in a 96-well plate (#167008, Thermo Fisher Scientific) format. For each well, 7500 LN229 cells were seeded (23,500/cm^2^) in the presence of compound dilutions or a DMSO control, with a final volume of 200 μL per well. To perform the assay, 22 μL of PrestoBlue HS reagent (representing a 10% volume addition) was added to each well under low-light conditions. The plate was then covered with aluminum foil and incubated at +37 °C for 60 min. For background control, the PrestoBlue reagent was diluted 1:10 in growth medium and incubated under identical conditions, protected from light. Following incubation, 100 μL of medium from each PrestoBlue-treated well was transferred to a new well. Prior to transfer, the medium was gently mixed by pipetting up and down four times to ensure homogeneity. 100 μL of the PrestoBlue background control mixture was also pipetted into new wells. The plate was centrifuged at 1200 rpm for 30 s to eliminate air bubbles. Fluorescence was measured using the PerkinElmer EnVision 2105 multimode plate reader, with excitation at 560 nm and emission detection at 590 nm

### Scratch Wound-Healing Assay

A total of 200,000 LN229 cells in 1 mL of growth medium were seeded (52,500/cm^2^) on 12-well plate (#CLS3513-50EA, Corning, USA) and incubated in +37 °C incubator for approximately 24 h or until the wells were confluent. When the wells were deemed confluent, they were washed with PBS and immersed in 1 mL of treatment medium containing either DMSO or compound diluted in 1% FBS growth medium, and the plate was incubated in a +37 °C incubator for 24 h. After the 24-h preincubation period, a horizontal line was marked on the bottom of the wells to aid later imaging. Using a 200 μL pipet tip, attached to a pipet, a scratch wound was done perpendicular to the horizontal line on the bottom of the well. The wells were then washed two times with PBS and the same dilution of DMSO or compound treatment in 1% FBS growth medium as in the previous 24-h incubation period was applied to the wells. Scratch wounds were then immediately imaged with an Olympus (Tokyo, Japan) inverted microscope with a Thorlabs (Newton, USA) camera. The plate was put into a +37 °C incubator and images of scratch wounds were taken after 24- and 48-h of incubation. Images of scratch-wound closure were quantified using ImageJ software with a modified previously established macro for the scratch wound healing assay.^49^

### Transwell Invasion Assay

To perform Transwell invasion assays, Millicell 24-well inserts with 8.0 μm membrane pores (#PTEP24H48, Merck Millipore, Burlington, USA) were coated with 100 μL of growth factor-reduced Matrigel (#354230, LOT 19224001, Corning) at a 0.3 mg/mL concentration and incubated at +37 °C for 1 h. During incubation of the coated inserts , LN229 cells were detached and further diluted to 0.75 × 10^6^/mL concentration in serum-free medium. After incubation, the Matrigel was carefully suctioned from the wells, and 200 μL of the cell suspension was loaded into the inserts. Inserts were applied in 24-well plate wells, and 600 μL of normal 10% FBS growth medium was loaded into the bottom of each well for chemoattraction. Inserts were incubated for approximately 22 h at +37 °C, after which the remaining cell suspension and noninvaded cells were carefully removed from the inserts with cotton-tipped swabs. Inserts were then first put into new wells with 1× PBS, from where they were transferred to wells containing 70% ethanol for 10–15 min at RT to fix the invaded cells. After fixation, inserts were allowed to dry before staining with 0.2% crystal violet for 3–5 min at RT. Stained inserts were thoroughly washed with PBS to remove any excess crystal violet. Inserts were allowed to dry, and the membranes were carefully detached from the inserts with a surgical scalpel. Membranes were applied to microscope slides and imaged with a Leica inverted microscope. For data quantification, five randomly selected fields per insert were imaged at 10× magnification, and the average number of invaded cells was quantified using ImageJ software.

### Mouse Cortical Neuron Culture and Viability Assay

Mouse primary cortical neurons were cultured as previously reported.^50^ Brain cortices were dissected from E15.5 embryos and incubated with 0.5% trypsin (103139, MP Biomedicals) for 20 min at 37 °C, and then mechanically dissociated. 6 × 10^3^ cells were plated in the center of 96-well plates coated with poly-l-ornithine (P-8638, Sigma-Aldrich) and cultured in neurobasal medium (21103–049, Invitrogen/Gibco) containing B27 supplement (17504-001, Invitrogen/Gibco) and 0.5 mM l-glutamine (25030-032, Invitrogen/Gibco) under standard culture conditions (5% CO2, 37 °C). For the viability assay, cells were cultured for 5 days and then treated with the compounds for 3 days. Neuronal cultures were then fixed and stained with anti-NeuN antibodies (1:1000 dilution; MAB377, Sigma-Aldrich, Germany). Images were acquired by ImageXpress Nano high-content imaging equipment. Immunopositive neurons were counted by CellProfiler software, and the data were analyzed by CellProfiler Analyst software. The results are expressed as the percentage of cell survival compared with nontreated neurons.

### Patents

The following two patents have been submitted:

1. Method of suppressing cancer by inhibitors of RNA m^6^A YTHDF family binding proteins (Appl. No. PCT/EP2025/065233). Assignee: Chemestmed, Ltd. Inventors: Esko Kankuri, Vilbert Sikorski, Simona Selberg, Larisa Ivanova, Neinar Seli, and Mati Karelson.
2. Method of suppressing cancer by 3-alkyl-2-sulfanylidene-1H-thieno­[2,3-*d*]­pyrimidin-4-one derivatives inhibiting RNA m^6^A YTHDF family binding protein YTHDF2. (Appl. No. EP26193272.7). Assignee: Chemestmed, Ltd. Inventors: Esko Kankuri, Vilbert Sikorski, Joni Salo, Simona Selberg, Larisa Ivanova, Neinar Seli, and Mati Karelson.

## Supplementary Material



## Notes

Table S1: Docking efficacies of tool compounds that showed activity in *in vitro* experiments for YTHDF1–3 m^6^A binding inhibition (relates to Figures 2–3); Table S2: Docking efficacies of the commercially available derivative compounds for DT4 and DT13, which showed first activity in *in vitro* experiments for YTHDF1–3 m^6^A binding inhibition (relates to Figure 2); Figure S1: Binding-induced chemical shift perturbations on ^15^N YTHDF2 m^6^A binding YTH-domain upon addition of DT11 at high concentrations (relates to Figure 6) (PDF)
