# Supplementary material for: Discovery and Design of YTHDF2-Selective and Pan-YTHDF Inhibitors for m6A Binding by the YTH Aromatic Cage
Source: ACS Omega. 2026 Oct 14;11(42):64760–78. doi: 10.1021/acsomega.6c05268 (PMC13717755; doi:10.1021/acsomega.6c05268)
Supplement: Supplementary file 1 [file ao6c05268_si_001.pdf]

Supporting Information for Publication

Discovery and design of YTHDF2-selective and pan-YTHDF inhibitors for m^6A binding by the YTH aromatic cage

Vilbert Sikorski M.D.,[a,b,*] Joni Salo MSc.,[a] Simona Selberg Ph.D.,[c,d] Diogo Costa Sa,[a] Brunaldo Renzi Msc.,[e] Indrek Teino, Ph.D.,[e] Daria Blokhina MSc,[a] Salla Virtanen Ph.D.,[e] Lina Antenucci Ph.D.,[f,g] Mart Saarma Ph.D.,[e] Perttu Permi Ph.D.,[e,f,g] Mati Karelson Ph.D.,[c,d] Esko Kankuri M.D. Ph.D.[a]

[a] Department of Pharmacology, Haartmaninkatu 8, 00290, University of Helsinki, Helsinki, Finland.
[b] Heart and Lung Center, Haartmaninkatu 4, 00290, Helsinki University Hospital, Helsinki, Finland
[c] Institute of Chemistry, Ravila tn 14a, 50411, University of Tartu, Tartu, Estonia
[d] Chemestmed, Ltd, Soola tn 8, 51014, Tartu, Estonia
[e] Institute of Biotechnology, HiLIFE, Viikinkaari 5d, 00790, University of Helsinki, Helsinki, Finland.
[f] Department of Chemistry, Nanoscience Center, Survontie 9 C, 40500, University of Jyväskylä, Finland.
[g] Department of Biological and Environmental Science, Nanoscience Center, Survontie 9 C, 40500, University of Jyväskylä, Finland.

[*] Corresponding author

Corresponding author details: Vilbert Sikorski MD, Faculty of Medicine, Department of Pharmacology, PO Box 63 (Haartmaninkatu 8), FIN-00014 University of Helsinki, Finland. Email vilbert.sikorski@helsinki.fi, tel. +358-50-919-0914.

Table S1.
Docking efficacy (DE) values of compounds binding to YTHDF1 (DF1), YTHDF2 (DF2) and YTHDF3 (DF3) proteins that showed activity in *in vitro* experiments.

No.	Compound structure	DE (DF1)	DE (DF2)	DE (DF3)
DT4	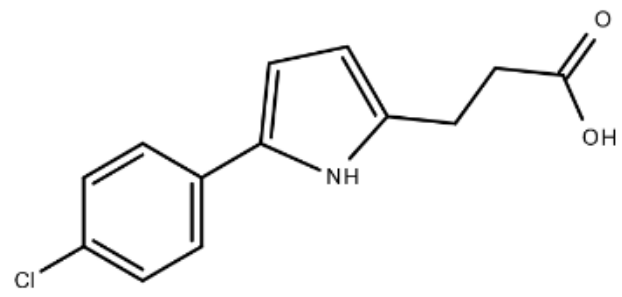	0.338	0.287	0.362
DT6	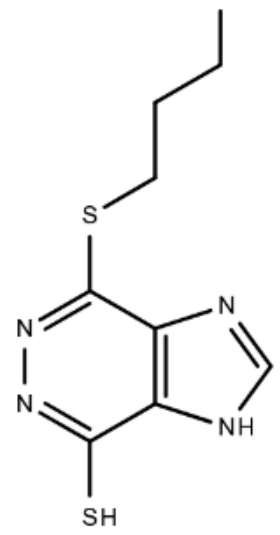	0.47	0.419	0.463
DT10	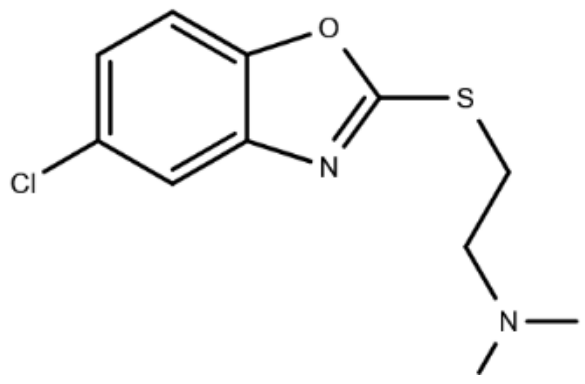	0.373	0.436	0.385
DT11	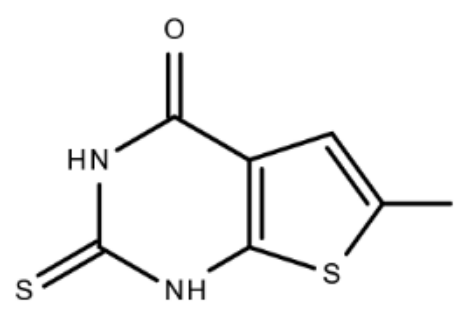	0.617	0.594	0.633
DT13	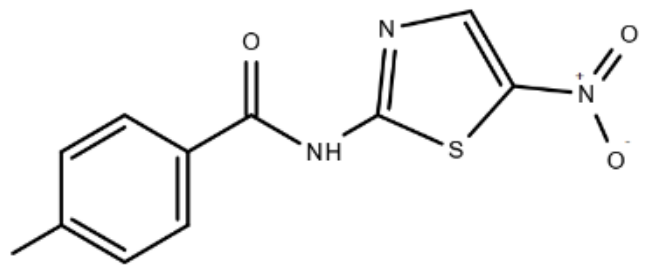	0.341	0.402	0.314
DT14	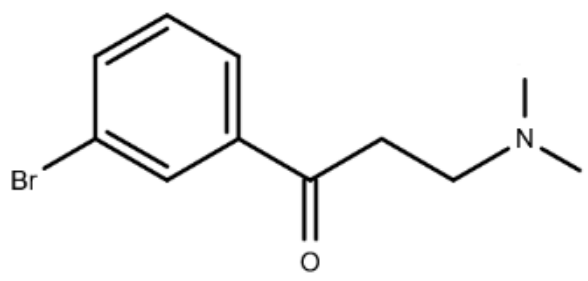	0.476	0.500	0.523
DT15	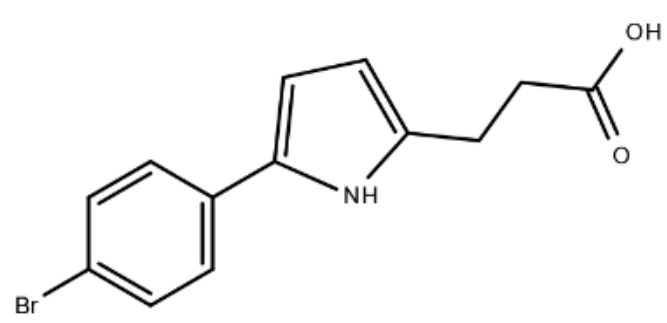	0.336	0.299	0.368

Table S2.
Docking efficacy (DE) values of the binding of compounds analogous to compounds DT4 and DT13 to YTHDF1 (DF1), YTHDF2 (DF2) and YTHDF3 (DF3) proteins.

No.	Compound structure	DE (DF1)	DE (DF2)	DE (DF3)
DT4-1	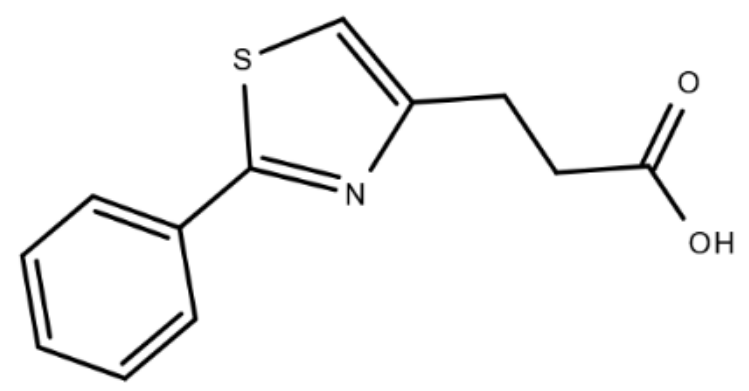	0.364	0.307	0.344
DT4-2	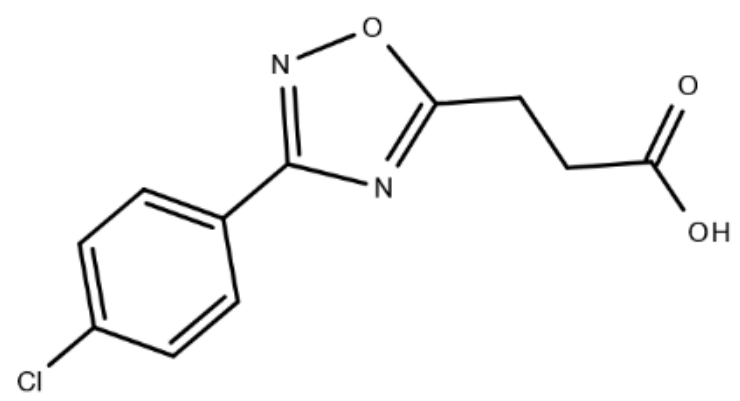	0.368	0.289	0.357
DT13-1	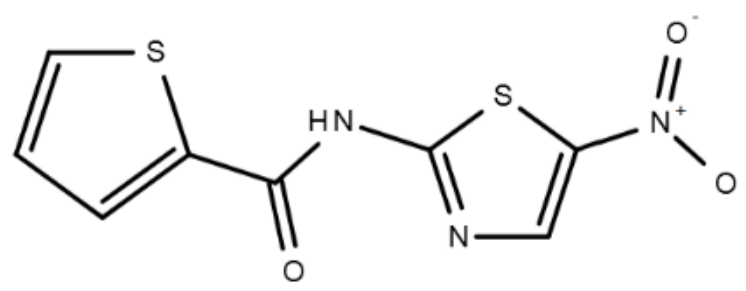	0.316	0.291	0.303
DT13-2	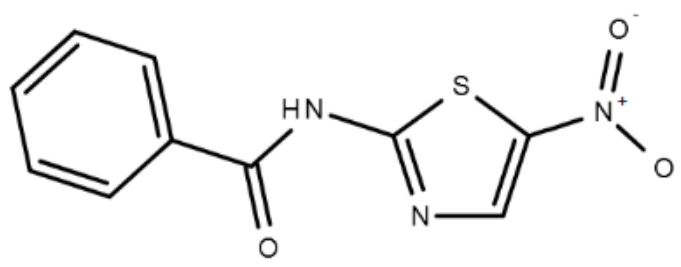	0.25	0.276	0.321
DT13-3	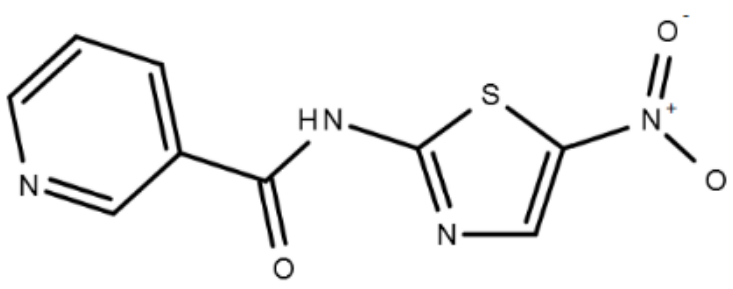	0.317	0.301	0.286
DT13-4	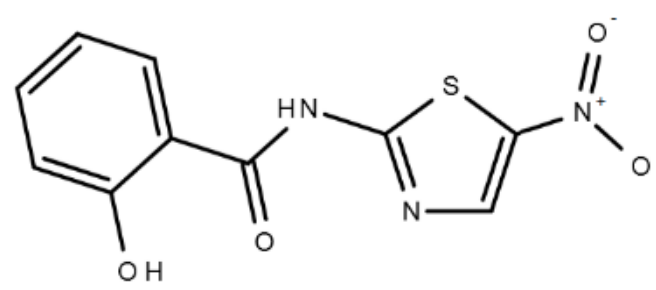	0.316	0.322	0.317
DT13-5	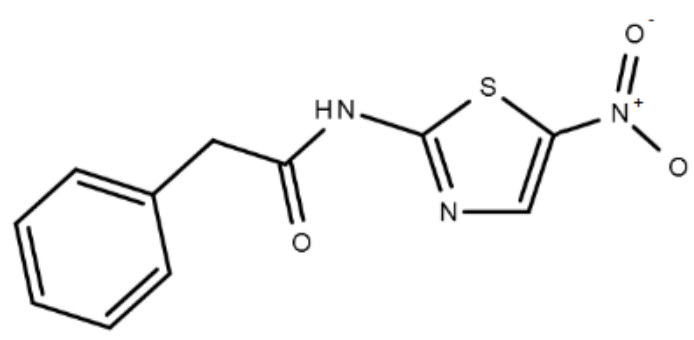	0.454	0.26	0.296

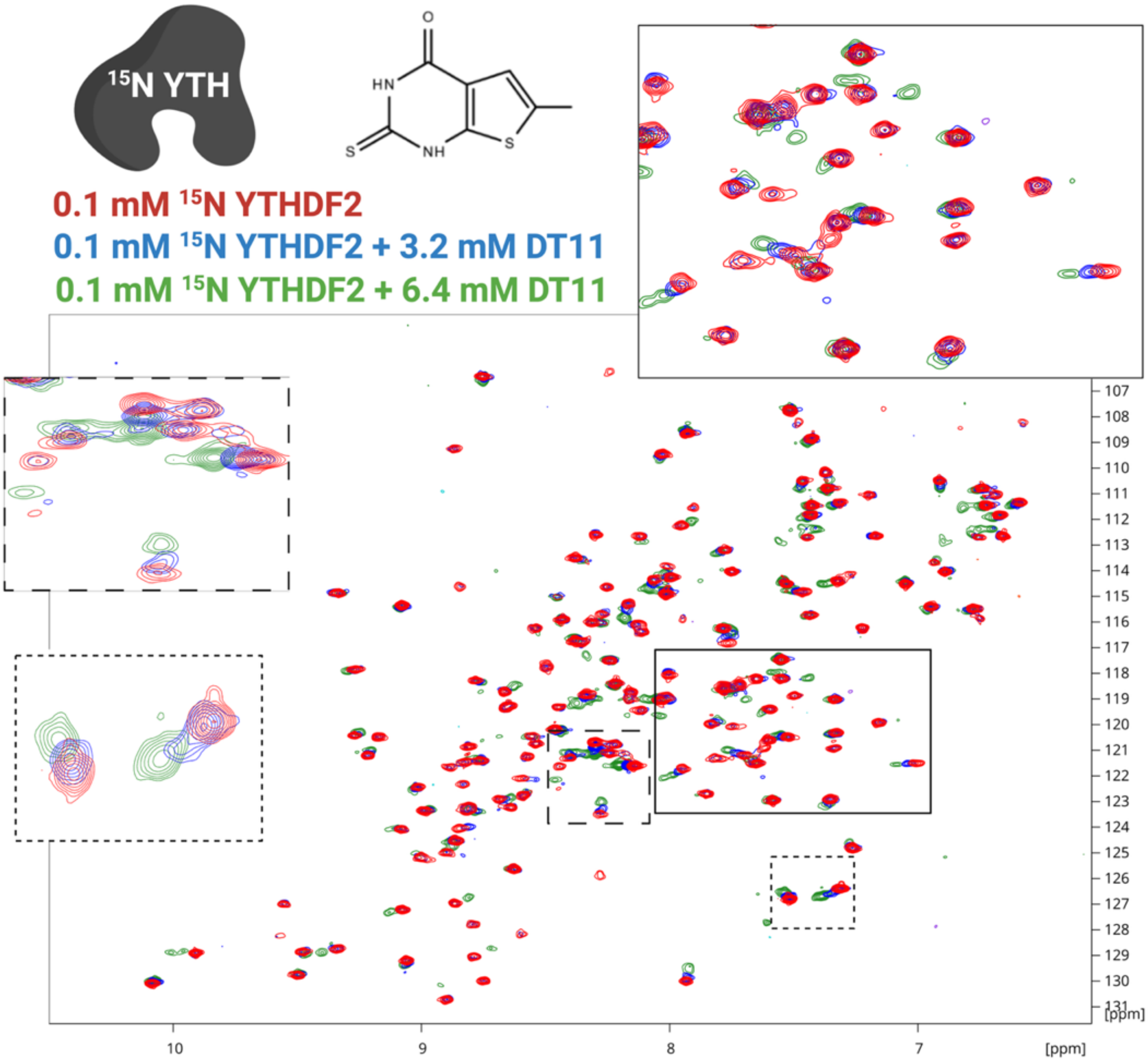

Figure S1.

Binding induced chemical shift perturbations on ^{15}N YTHDF2 m^{6}A binding YTH-domain upon addition of DT11 at high concentrations. ^{15}N-HSQC spectra of free 0.1 mM ^{15}N-labeled YTHDF2 (red), and in the presence of 3.2 mM (blue) and 6.4 mM (green) of unlabeled DT11, are shown overlaid. Three insets, indicated by solid and dashed line rectangular, showcase expansions from selected spectral regions, where significant chemical shift perturbations (CSPs) can be observed. Prior to the final titration point (6.4mM of DT11), rather small CSPs take place, agreeing to specific interaction, but at 64x, approximately half of the YTHDF2 residues undergo CSPs, suggesting, beyond specific binding between YTHDF2 and DT11, possible phase shift or oligomerization effects.
